# Analysis of Protein Palmitoylation Reveals a Pervasive Role in *Plasmodium* Development and Pathogenesis

**DOI:** 10.1016/j.chom.2012.06.005

**Published:** 2012-08-16

**Authors:** Matthew L. Jones, Mark O. Collins, David Goulding, Jyoti S. Choudhary, Julian C. Rayner

**Affiliations:** 1 Malaria Programme, The Wellcome Trust Sanger Institute, The Wellcome Trust Genome Campus, Hinxton, Cambridge CB10 1SA, UK; 2 Proteomic Mass Spectrometry, The Wellcome Trust Sanger Institute, The Wellcome Trust Genome Campus, Hinxton, Cambridge CB10 1SA, UK; 3 Microbial Pathogenesis, The Wellcome Trust Sanger Institute, The Wellcome Trust Genome Campus, Hinxton, Cambridge CB10 1SA, UK

## Abstract

Asexual stage *Plasmodium falciparum* replicates and undergoes a tightly regulated developmental process in human erythrocytes. One mechanism involved in the regulation of this process is posttranslational modification (PTM) of parasite proteins. Palmitoylation is a PTM in which cysteine residues undergo a reversible lipid modification, which can regulate target proteins in diverse ways. Using complementary palmitoyl protein purification approaches and quantitative mass spectrometry, we examined protein palmitoylation in asexual-stage *P. falciparum* parasites and identified over 400 palmitoylated proteins, including those involved in cytoadherence, drug resistance, signaling, development, and invasion. Consistent with the prevalence of palmitoylated proteins, palmitoylation is essential for *P. falciparum* asexual development and influences erythrocyte invasion by directly regulating the stability of components of the actin-myosin invasion motor. Furthermore, *P. falciparum* uses palmitoylation in diverse ways, stably modifying some proteins while dynamically palmitoylating others. Palmitoylation therefore plays a central role in regulating *P. falciparum* blood stage development.

## Graphical abstract

**Graphical Abstract:**

**Figure:**
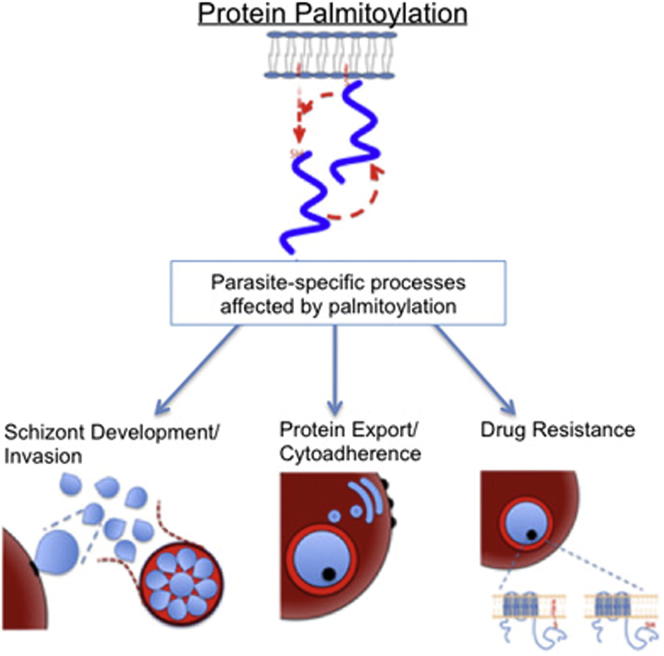

**Highlights:**

► A global approach identified >400 palmitoylated proteins in *Plasmodium falciparum* ► Palmitoyl proteins are central to invasion and other virulence-associated processes ► Palmitoylation is required for completion of the *P. falciparum* asexual life cycle ► *P. falciparum* uses palmitoylation dynamically for diverse regulatory purposes

## Introduction

*Plasmodium falciparum* is responsible for almost all malaria-induced mortality and is a major threat to global public health (Murray et al., 2012; Snow et al., 2005). While it has a complex life cycle, it only causes disease while undergoing development and asexual multiplication within human erythrocytes—where it alters its host cell to support nutrient uptake, reproduction, and immune evasion (Miller et al., 2002). Asexual-stage development is tightly regulated, and large-scale studies of transcription have emphasized a key role for gene regulation in controlling this process (Bozdech et al., 2003; Lasonder et al., 2002; Otto et al., 2010). Protein posttranslational modifications (PTM) are also commonly used to regulate biological processes, and should be fundamental to the coordination of *P. falciparum* development. In recent years, the advent of specific purification methods and highly sensitive mass spectrometry approaches have allowed large-scale analyses of PTMs such as protein phosphorylation, ubiquitination, and acetylation and have provided valuable insight into protein regulation (Choudhary et al., 2009; Rigbolt et al., 2011; Xu et al., 2010). These approaches are only now being applied to *P. falciparum*, but, as the recent publication of the *P. falciparum* phosphoproteome illustrates, they will provide important insight that supports the generation of diverse hypotheses probing key aspects of parasite biology (Treeck et al., 2011).

Protein palmitoylation, the reversible addition of a 16-carbon saturated fatty acid to cysteine residues, is a PTM that regulates protein localization, activity, and membrane binding (Linder and Deschenes, 2007; Resh, 2006a). For example, targeting of H/Nras GTP-binding proteins to the plasma membrane, Golgi network, or endoplasmic reticulum depends on its dynamic palmitoylation/depalmitoylation (Rocks et al., 2005). For dynamically palmitoylated proteins like these, the addition and removal of palmitoyl groups can be rapid and tightly regulated, while for other proteins, palmitoylation can be stable and exert a long-term effect (Resh, 2006a). While there are a few loosely defined palmitoylation motifs, such as a cysteine within three to four residues of an N-terminal myristoylation site, it is not generally possible to identify palmitoylated proteins by their primary sequence—a fact that has been reinforced by the development of proteome-level approaches for palmitoyl protein identification. These approaches have shown that palmitoylation is significantly more common than previously thought and affects a wide range of protein classes and cellular functions (Emmer et al., 2011; Kang et al., 2008; Martin and Cravatt, 2009; Roth et al., 2006; Wilson et al., 2011; Yang et al., 2010; Yount et al., 2010).

Protein palmitoylation in *P. falciparum* is poorly characterized, with only three experimentally confirmed palmitoyl proteins: PfGAP45, PfCDPK1, and Pf_calpain (Möskes et al., 2004; Rees-Channer et al., 2006; Russo et al., 2009). However, given the widespread use of palmitoylation as a regulatory mechanism in other eukaryotes, it is likely that *P. falciparum* uses palmitoylation extensively to regulate key aspects of its biology, including those central to pathogenesis. To determine the role of palmitoylation in regulating *P. falciparum* asexual-stage biology, we have characterized the palmitoylated portion of the schizont proteome using two complementary techniques: acyl-biotin exchange, and metabolic labeling with a click chemistry compatible palmitic acid analog (Martin and Cravatt, 2009; Wan et al., 2007). In order to confidently distinguish palmitoylated proteins from nonspecific background proteins, we used stable isotope labeling with amino acids in cell culture (SILAC) to allow highly accurate mass spectrometry-based protein quantification. The comparative use of these two independent purification strategies coupled with the use of SILAC to provide robust quantification allows for a thorough validation of identified palmitoyl proteins. Using these approaches, we have identified more than 400 putative palmitoyl proteins, including proteins essential for drug resistance, protein export, cytoadherence, schizont development, and invasion. This data set provides an important resource for the generation of hypotheses probing protein function and regulation in multiple areas of *P. falciparum* biology.

## Results

### Palmitoylated *P. falciparum* Proteins Can Be Purified with Both Acyl-Biotin Exchange and Metabolic Labeling/Click Chemistry

**Figure 1:**
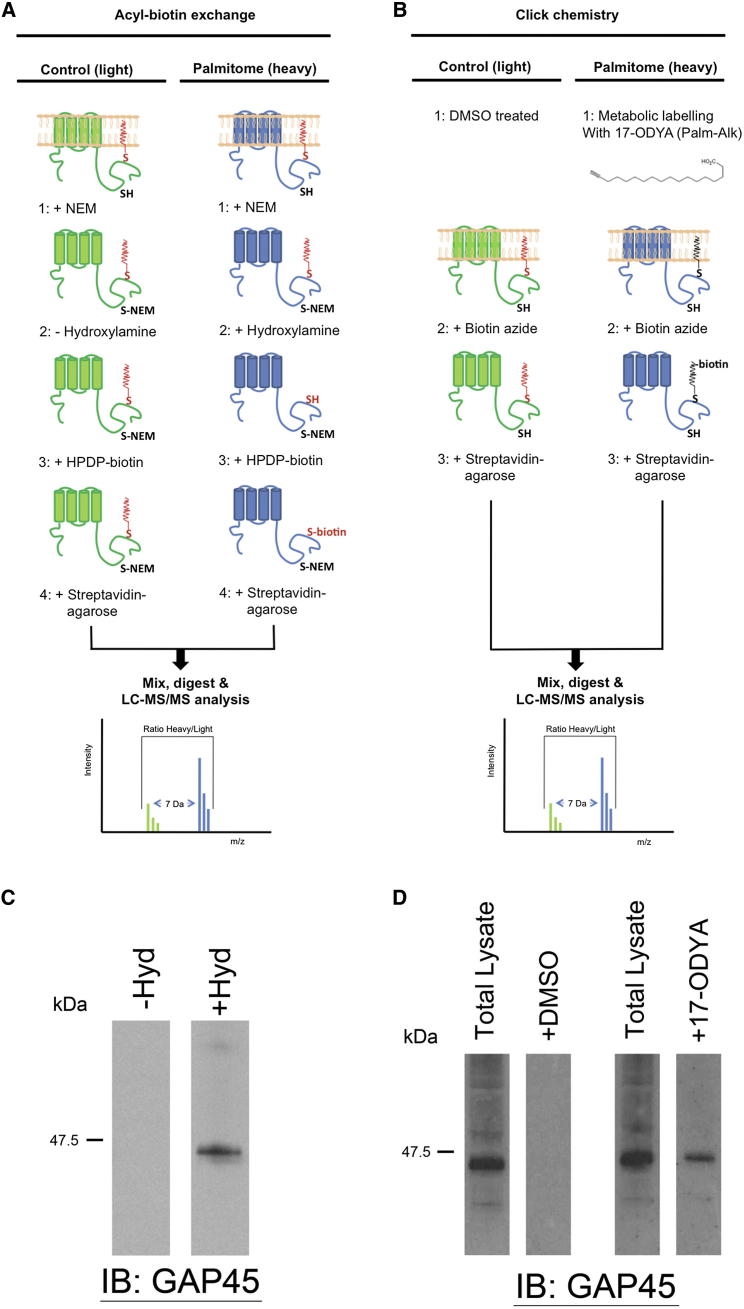
ABE and MLCC Allow Purification of a Known *P. falciparum* Palmitoyl Protein (A) An overview of the ABE-based strategy for mass spectrometry-based identification of palmitoyl proteins, as described in the main text. For SILAC labeling, parasites used to generate the mock hydroxylamine-treated control samples were gown in “light” amino acid-containing media, while parasites used for hydroxylamine treated samples were grown with a “heavy” amino acid. (B) An overview of the MLCC-based strategy for mass spectrometry-based identification of palmitoyl proteins, as described in the main text. For SILAC labeling, control parasites were grown in “light” amino acid-containing media, while 17-ODYA-labled parasites were grown with a “heavy” amino acid. (C) Anti-PfGAP45 antibodies were used to detect the presence of GAP45 in an ABE +hydroxylamine (palmitome; right lane) elution, and a mock hydroxylamine (control; left lane) elution. (D) Anti-PfGAP45 antibodies were used to detect the presence of GAP45 in a total 17-ODYA-labeled proteome (Total Lysate), and in a sample where click chemistry had been used to biotinylate and purify all 17-ODYA-labeled protein (palmitome; + 17-ODYA). Left-hand lanes show PfGAP45 present in total DMSO proteome (Total Lysate) and after a control click chemistry reaction (control; +DMSO). See also Figure S1.

Two fundamentally different strategies for the global purification of palmitoyl proteins have been reported: acyl-biotin exchange (ABE) and metabolic labeling with a palmitic acid analog followed by click chemistry (MLCC) (Martin and Cravatt, 2009; Roth et al., 2006). ABE allows the exchange of thioester-linked palmitoyl groups for a biotin moiety, which can then be used for specific purification of previously palmitoylated proteins (Figure 1B). For ABE, the total proteome is extracted, solubilized, and treated with N-ethylmaleamide (NEM), which irreversibly blocks free thiol groups on unmodified cysteines. Thioester bonds are then cleaved by hydroxylamine treatment, releasing S-linked palmitoyl groups to expose previously bound thiols, which are then covalently linked to HPDP-biotin. As a control for specificity, an equal quantity of the total proteome is subjected to ABE, but is not treated with hydroxylamine so palmitoyl moieties are not removed and palmitoylated proteins are not subsequently purified (see the Supplemental Experimental Procedures for a detailed description of the protocol).

MLCC is an orthogonal method of palmitome purification that relies on metabolic-labeling of cells with 17-octadecynoic acid, a palmitic acid analog with an alkyne group at the carbon-16 position. After 17-ODYA labeling, proteins are extracted and all analog-labeled proteins are irreversibly biotinylated using biotin-azide and copper (I)-catalyzed azide-alkyne cycloaddition (click chemistry), allowing these proteins to be purified on streptavidin-agarose (Figure 1D). MLCC has been used to characterize the palmitome from mammalian cell lines (Martin and Cravatt, 2009; Wilson et al., 2011; Yount et al., 2010), but has not previously been used in conjunction with ABE. It is important to note that while each of these methods allow robust palmitoyl protein purification, they will not isolate a completely overlapping set of proteins. For example, while ABE has the potential to capture the full palmitome, MLCC will only capture proteins that become palmitoylated during the metabolic labeling time course, and is therefore restricted to a more dynamic portion of the palmitome.

In order to show that each of these methods reproducibly purify palmitoyl proteins from *P. falciparum*, we probed palmitome eluates with antibodies specific to glideosome-associated protein 45 (PfGAP45), which is known to be palmitoylated. PfGAP45 was purified both by ABE and by MLCC, but was not detectable in control reactions for either method (Figures 1A and 1C). Importantly, treating 17-ODYA-labeled samples with hydroxylamine prevented purification of PfGAP45 by MLCC (Figure S1A available online). This establishes that PfGAP45 MLCC purification is dependent on the formation of a thioester bond, and provides direct evidence that 17-ODYA is used as a palmitic acid analog.

### Large-Scale Purification of *P. falciparum* Palmitoylated Proteins

**Figure 2:**
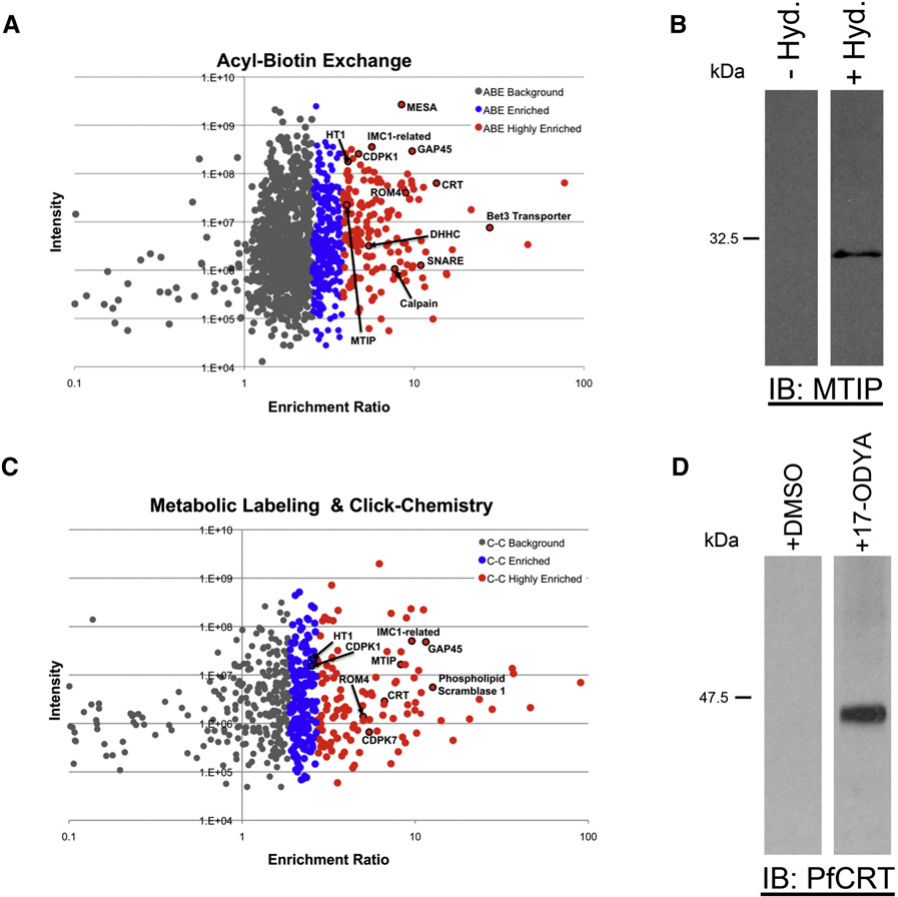
Large-Scale Identification of *P. falciparum* Palmitoyl Proteins (A) SILAC-based quantitative mass spectrometry of ABE-purified proteins, based on three biological replicates. Plots display median protein intensities (y axis) and MaxQuant-generated enrichment ratios (x axis) for all ABE-identified proteins. Proteins meeting criteria for designation as enriched (blue) or highly enriched (red) are noted. Proteins of particular interest are also identified by name. (B) Anti-PfMTIP antibodies were used to detect PfMTIP in ABE eluates to validate its identification by mass spectrometry. Immunoblots from a +hydroxylamine (palmitome; right lane) eluate and –hydroxylamine (control; left lane) eluate were probed with anti-PfMTIP immune sera. (C) SILAC-based quantitative mass spectrometry of *P. falciparum* proteins purified by MLCC, based on two biological replicates. Plots display median protein intensities (y axis) and MaxQuant-generated enrichment ratios (x axis) for all MLCC-identified proteins. Proteins meeting criteria for designation as enriched (blue) or highly enriched (red) are noted. Proteins of particular interest are also identified by name. (D) Anti-PfCRT antibodies were used to detect PfCRT in MLCC eluates to validate its identification by mass spectrometry. Immunoblots from a (+)17-ODYA (palmitome; right lane) eluate and (+)DMSO (control; left lane) eluate were probed with anti-PfCRT immune sera. See also Figure S2 and Tables S1 and S2.

We performed large-scale ABE and MLCC from *P. falciparum* schizont stage parasites, with three biological replicates purified with ABE and two with MLCC. A small aliquot of the eluates from each replicate was assayed for PfGAP45 enrichment before mass spectrometry analysis, and PfGAP45 was clearly enriched in all palmitome fractions but was not detectable in any control fraction (Figure S2). All samples were SILAC labeled during in vitro culture to allow for quantitative assessment of palmitoyl protein enrichment in palmitome versus control elutions (Figures 1B, 1D, 2A, and 2C). High-resolution tandem mass spectrometry analysis of pooled (heavy and light SILAC-labeled), gel-separated palmitome, and control elutions was performed and MaxQuant (Cox and Mann, 2008) was used to generate specific ratios of enrichment (palmitome over control), which were used to classify putative palmitoyl proteins and to test the reproducibility of each method (Figure S2). All proteins given an enrichment ratio by MaxQuant were subsequently grouped into background, enriched, or highly enriched classes based both on their magnitude of enrichment and on the robustness of their ratio assignments (Tables S1 and S2; see the Supplemental Information for a complete description of the cutoff criteria).

**Figure 3:**
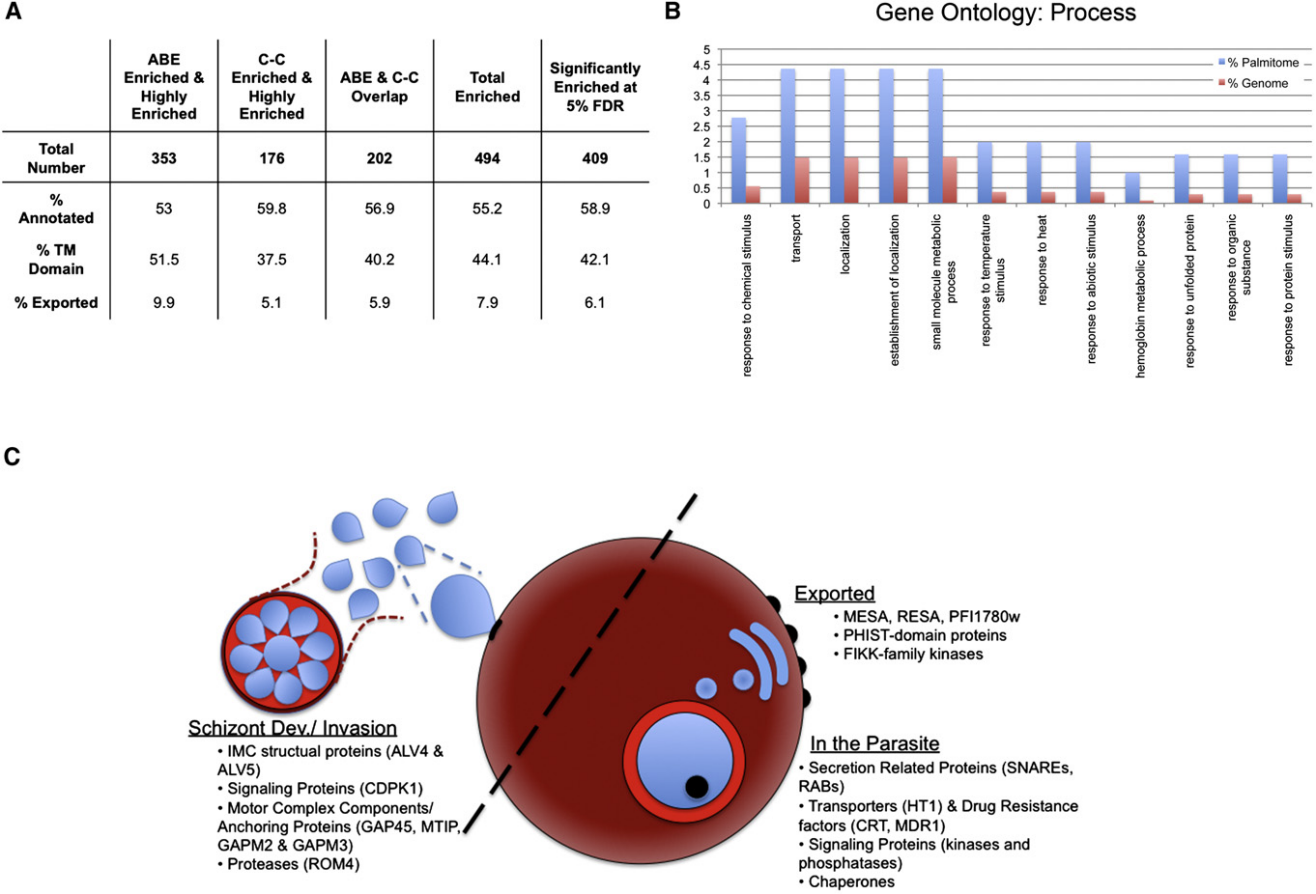
The *P. falciparum* Palmitome (A) Total palmitoyl protein identifications resulting from this work broken down by purification method and with corresponding percentages of annotated, TM domain-containing, and exported proteins. (B) Gene Ontology analysis of total enriched proteins. GO analysis is of “biological process” annotations and is presented in comparison to corresponding percent genome values. p values are ≤ 0.05 for all displayed terms. (C) Selected *P. falciparum* palmitoyl proteins important for schizont development and invasion, protein export, and intraparasitic processes. See also Figure S3 and Tables S3, S4, and S5.

In total, 1,752 proteins were identified from late asexual-stage *P. falciparum* at a false discovery rate of 1%. With both ABE and MLCC, we observed a wide range of enrichment values across the proteome (Figures 2A and 2C). Critically, with each method a large number of proteins were not significantly enriched, highlighting the importance of robust quantitative analysis to allow for distinction between enriched palmitoyl proteins and nonspecific proteins (Figures 2A and 2C). With each palmitoyl protein purification method we also observed a wide dynamic range of protein intensity values, indicating excellent depth of proteome coverage, though ABE clearly produced the more complex sample (Figure 2A). The greater complexity of the ABE output is also reflected in the larger number of significantly enriched proteins identified by this method (Figure 3A).

Importantly, each method displayed strong enrichment of known palmitoyl proteins (PfGAP45 and Pf_calpain; Figures 2B and 2D). Proteins known to be palmitoylated in other systems (Bet3 transporter, phospholipid scramblase 1, SNAREs, for example; Figures 2A and 2C) were also identified as enriched, providing further validation of each purification method and for our assignment of enrichment classes. To give a visual representation of relative enrichment levels, palmitome and control eluates from each method were probed with antibodies against PfMTIP and PfCRT—proteins that have been highly studied but that have not previously been identified as palmitoylated. Immunoblot data clearly validates assignment of these proteins as palmitoyl proteins (Figures 2B and 2D).

### ABE- and Click Chemistry-Based Palmitome Purification Methods Are Complementary

The palmitome purification strategies outlined above are robust and well validated; however, it is important to note that each method will result in the isolation of unique classes of false positives. ABE, since it relies on complete NEM-blockage of all free thiols, can result in the enrichment of highly abundant proteins where thiol blockage may not proceed to completion. ABE also reproducibly purifies enzymes that use thioester-linked acyl intermediates in their reaction mechanism (ubiquitin ligases, for example [Yang et al., 2010]). MLCC results in the enrichment of non-palmitoyl proteins that have incorporated 17-ODYA but that are not S-acylated. *P. falciparum* GPI-anchors, for example, have a palmitoyl-linked inositol ring (Naik et al., 2000), resulting in the reproducible purification of GPI-linked proteins after MLCC. Since these methods have unique classes of false positives, we have analyzed all resulting data in two ways: first, we have created enriched and highly enriched classes for each individual method, and second we have taken advantage of the complementary nature of the two approaches to create a group of putative palmitoyl proteins identified as enriched by *both* methods. For this group of overlapping identifications, we relaxed protein abundance cutoffs to avoid eliminating lower abundance proteins that might not be seen in all five replicates across the two purification strategies (see the Supplemental Experimental Procedures for full enrichment cutoff details).

ABE resulted in the identification of 111 highly enriched proteins and a further 242 proteins classified as enriched (Figure 3A and Table S1). MLCC resulted in the identification of 82 highly enriched proteins and 94 enriched proteins (Figure 3A and Table S2), which again reflects the lower complexity of the MLCC samples. Using the combined approach, 202 proteins were enriched by *both* methods (Figure 3A and Table S3). Strikingly, 57.2% of the total enriched fraction identified by MLCC is enriched by ABE. This level of coenrichment is significantly higher than the highest previously seen when separate palmitome reports using only one or the other method are compared (35% [Wilson et al., 2011]).

### The *P. falciparum* Palmitome

After analysis of all enrichment data, it is apparent that ABE and MLCC isolate both overlapping and unique portions of the *P. falciparum* palmitome (Tables S1, S2, and S3), as has been reported when comparing phosphoproteome purification techniques (Bodenmiller et al., 2007). With each method, we have not observed a striking bias in enrichment of Gene Ontology (GO) terms or specific protein classes that would predictably explain variations in these data sets, although, again, some differences are likely due to MLCC only capturing proteins that have incorporated 17-ODYA during the labeling time course. We have subsequently compiled all proteins classified as enriched by any of our cutoff criteria into one list of 494 putative *P. falciparum* palmitoyl proteins (Figure 3A and Table S4). While this set of 494 proteins may contain false positives, we consider this the closest approximation of the *total* palmitome from *P. falciparum* schizonts. However, as a final layer of validation, we performed statistical analysis of both ABE and MLCC data sets together and 409 of the 494 proteins designated as enriched by our criteria were confirmed as significant by this analysis (using a 5% false discovery rate and t test with Benjamini and Hochberg correction for multiple hypothesis testing; Table S4). Some of the proteins that did not reach significance did not have at least three replicate ratios across the five ABE and MLCC experiments, which was required for this analysis (for total enrichment data used to determine cutoffs and compile the *P. falciparum* palmitome, see Table S5).

GO analysis of the total *P. falciparum* palmitome reveals enrichment of proteins involved in transport, establishment of localization, and in stimulus response, which is consistent with palmitome GO analyses in other systems (Figure 3B) (Wilson et al., 2011). A selected set of 55 proteins enriched by both ABE and MLCC are presented in Table 1, and this list includes scaffolding, cytoskeletal, adhesion, metabolism-related, chaperone, signaling, and transport proteins, all of which are protein classes identified as palmitoylated in other organisms. While GO analysis implies a significant conservation of *Plasmodium* palmitoyl protein function, it is also apparent that many *P. falciparum* palmitoyl proteins are involved in essential *parasite-specific* processes (Figure 3C). Examples include proteins involved in drug resistance (PfCRT, PfMDR1), schizont development (ALV4, ALV5), erythrocyte invasion (PfMTIP, PfGAPM3, PfROM4), and protein export (PfMESA, PfRESA).

### Protein Palmitoylation Is Essential for Schizont Development and Is Directly Involved in Regulating Erythrocyte Invasion

**Figure 4:**
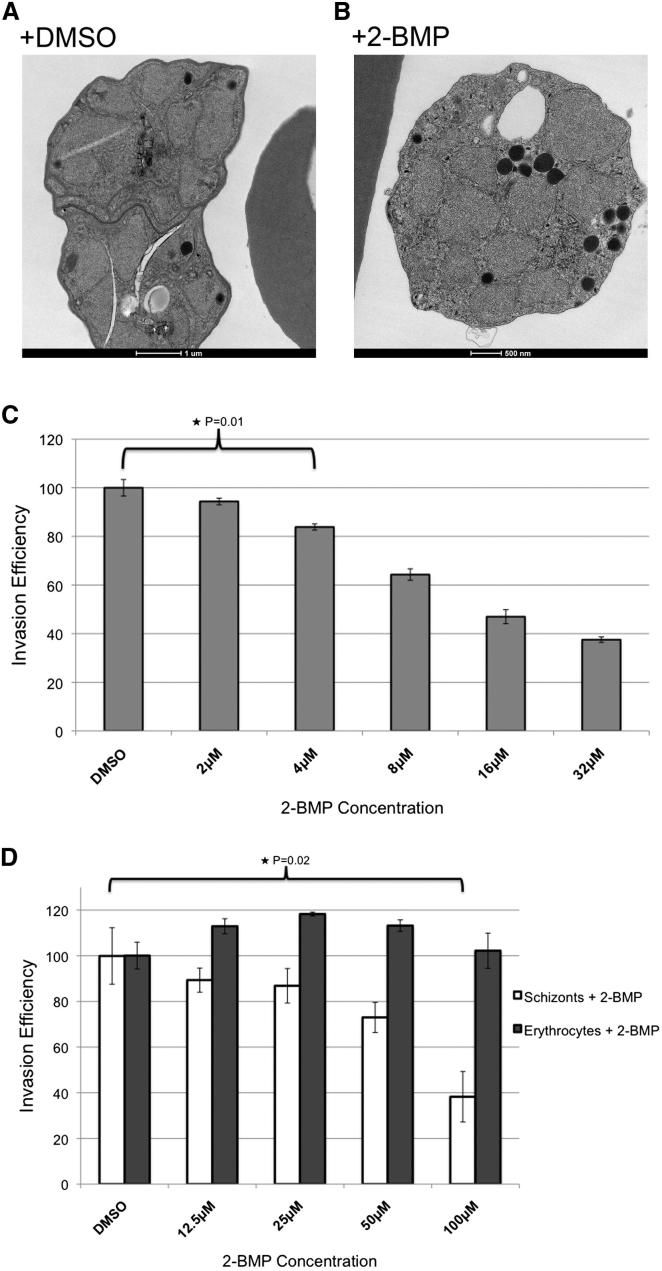
Palmitoylation Is Essential for Schizont Development and Invasion (A) Electron micrograph of mature schizonts (45–48 hr after invasion) after 24 hr treatment with DMSO. (B) Electron micrograph of mature schizonts (45–48 hr after invasion) after 24 hr treatment with 100 μM 2-BMP. (C) 2-BMP treatment inhibits erythrocyte invasion at low concentrations. Erythrocyte invasion efficiency was measured after treatment with increasing concentrations of 2-BMP. The histogram reports results from three biological replicates, and error bars indicate the SEM. p values were calculated with a student’s t test. (D) 2-BMP inhibits erythrocyte invasion by affecting *P. falciparum*-specific processes. Invasion efficiency was measured after mixing of 2-BMP-treated schizonts with DMSO-treated erythrocytes (white bars) or 2-BMP-treated erythrocytes with DMSO-treated schizonts (gray bars). The histogram reports the results of three biological replicates, and error bars indicate the SEM. p values were calculated with a student’s t test.

The *P. falciparum* palmitome includes a significant number of proteins important for schizont maturation and invasion, implying a role for palmitoylation in these processes (Figure 3C). To gain insight into the regulatory role of palmitoylation in these processes, we have used 2-bromopalmitate (2-BMP), an inhibitor of palmitoylation (Resh, 2006b), to disrupt palmitoylation in *P. falciparum*. First, we treated late trophozoite/early schizont-stage parasites with 100 μM 2-BMP (a 2-BMP concentration typically used to functionally inhibit palmitoylation in other systems [Resh, 2006b]) and examined their ultrastructure approximately 24 hr after treatment (Figures 4A and 4B). DMSO-treated control parasites developed normally, with near fully formed merozoites visible within late segmented schizonts (Figure 4A). 2-BMP treated parasites, however, developed abnormally, with severe disruption of intracellular membranes (Figure 4B). Strikingly, 2-BMP, in addition to generally disrupting cellular membranes, caused a gross mislocalization of rhoptries (Figure 4B). In DMSO treated parasites, single rhoptry structures are located within single developing merozoites, while in 2-BMP-treated parasites rhoptries have formed but are in aggregates or are not enclosed within a specific parasite structure (Figures 4A and 4B).

This phenotype indicated a possible role for palmitoylation in completing asexual-stage development. We therefore treated late schizonts with increasing concentrations of 2-BMP and observed that erythrocyte invasion is significantly inhibited by low 2-BMP doses (Figure 4C). To rule out an indirect effect through an impact on host erythrocytes, we repeated these assays using a two-color flow cytometry-based approach that allows separate treatment of late schizonts or target erythrocytes (Theron et al., 2010). Treatment of parasites with 2-BMP again resulted in a decrease in invasion, albeit with a lower degree of sensitivity, presumably because parasites were treated for only 4 hr before being thoroughly washed and mixed with erythrocytes. By contrast, treatment of erythrocytes with 2-BMP and mixing them with mock-treated parasites had no impact on invasion, demonstrating that the effect of 2-BMP is parasite specific (Figure 4D).

### Protein Palmitoylation Directly Regulates the Stability of Invasion Motor Components PfGAP45 and PfMTIP

**Figure 5:**
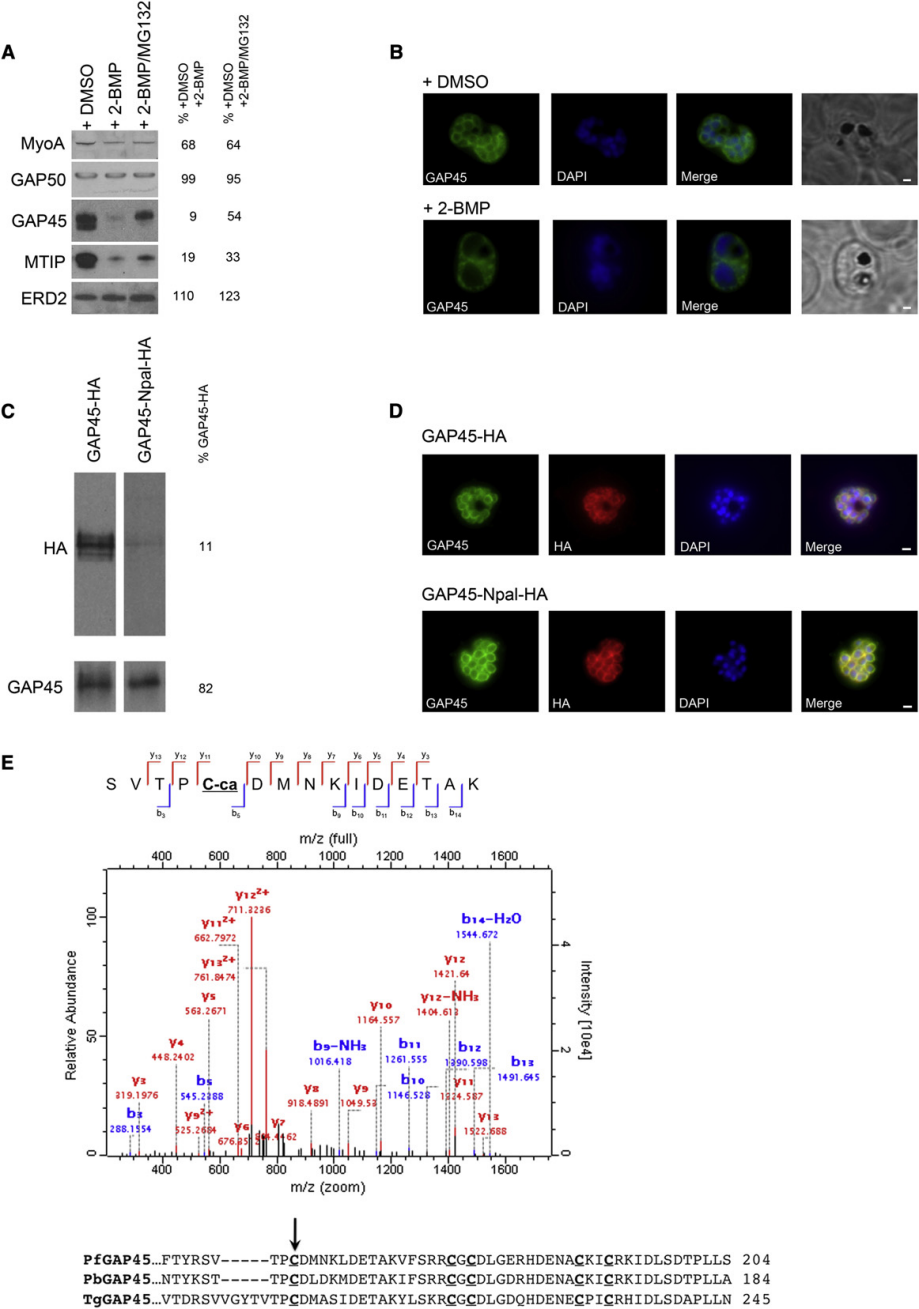
Palmitoylation Directly Affects Specific Invasion Motor Complex Components, and the PfGAP45 C-Terminal Domain Is Palmitoylated (A) Effect of 2-BMP on invasion motor protein levels. Saponin lysates from *P. falciparum* treated with 50 μM 2-BMP, cotreated with 50 μM 2-BMP and 20 μM of proteasome inhibitor MG-132, or treated with DMSO were probed with antibodies to PfGAP45, PfGAP50, PfMTIP, PfMyoA, or PfERD2 as a loading control. Changes in protein level were quantified by densitometry and are presented as a percentage of corresponding expression levels in DMSO treated extracts. (B) Effect of 2-BMP on PfGAP45 localization. Late schizonts (44–48 hr after invasion) were treated with 50 μM 2-BMP, and PfGAP45 targeting to the inner membrane complex (IMC) was assessed with anti-PfGAP45 antibodies in immunofluorescence assays. DMSO treated parasites were used as a control. Scale bars represent 1 μM. (C) Mutation of the PfGAP45 N-terminal palmitoylation site (cys5) affects PfGAP45 expression. Saponin lysates from schizont stage parasites expressing native (PfGAP45-HA) or mutant (PfGAP45-Npal-HA) protein were probed with an anti-HA monoclonal and with anti-PfGAP45 immune sera as a loading control. Quantification of PfGAP45-Npal-HA loss is presented as a percentage of PfGAP45-HA expression. (D) Mutation of the PfGAP45 N-terminal palmitoylation site does not prevent IMC targeting. Anti-HA and anti-PfGAP45 antisera were used to follow localization of tagged and endogenous GAP45 by immunofluorescence in late schizont stage PfGAP45-HA and PfGAP45-Npal-HA expressing parasites. Scale bars represent 1 μM. (E) PfGAP45 is palmitoylated at cysteine 160. MS/MS fragmentation spectrum of a peptide from PfGAP45 showing carbamidomethylation of cysteine 160. Alignment of the PfGAP45 C terminus with its Apicomplexan homologs show that cys160 is one of five well-conserved C-terminal domain cysteines. See also Figure S4.

Since palmitoylation is apparently essential for invasion, and because several invasion motor complex components are palmitoyl proteins, we next treated schizonts (approximately 42–44 hr after invasion) with 50 μM 2-BMP for 2–4 hr and examined its effect on specific complex members (Baum et al., 2006; Jones et al., 2006). Western blotting of 2-BMP-treated schizont material showed that levels of PfGAP45 and PfMTIP are severely reduced compared to DMSO-treated controls (Figure 5A). By contrast, complex components GAP50 and MyoA are unaffected. Treatment of parasites with a combination of 2-BMP and the proteasome inhibitor, MG-132, rescued a significant amount of PfGAP45, and, to a lesser extent, PfMTIP (Figure 5A). These results imply a direct relationship between palmitoylation of PfGAP45 and PfMTIP and protein stability, with each being degraded by the proteasome if palmitoylation is inhibited. Not surprisingly, the localization of the PfGAP45 remaining after 2-BMP treatment is disrupted, becoming more diffuse and losing any apparent IMC association (Figure 5B).

To more directly test the function of PfGAP45 palmitoylation, we generated *P. falciparum* lines expressing triple HA-tagged PfGAP45 (GAP45-HA) or PfGAP45 with its putative N-terminal palmitoylation site mutated to alanine (GAP45-Npal-HA) (Figure S3). These lines were made using the 3D7attB line (Nkrumah et al., 2006), and expression of GAP45-HA and GAP45-Npal-HA was in addition to that from the *PfGAP45* locus. Examination of GAP45-HA and GAP45-Npal-HA clearly shows a decrease in GAP45 expression after mutation of the N-terminal palmitoylation site (Figure 5C). This result replicates the effect of 2-BMP on endogenous PfGAP45 (Figure 5A) and provides additional evidence that 2-BMP specifically inhibits palmitoylation, though pleiotropic effects cannot be excluded.

In contrast to the effect of 2-BMP on PfGAP45 localization, the localization of the remaining GAP45-Npal-HA was unaffected, with the protein remaining at the IMC of developing merozoites, colocalized with endogenous PfGAP45 (Figure 5D). This is consistent with recently published results showing localization of *Toxoplasma gondii* GAP45 to the IMC after mutation of its N-terminal acylation motif. It was further shown that TgGAP45 acts as a link between the IMC and PM of tachyzoites, and that this linking function depends on conserved cysteine residues in its C-terminal domain that were hypothesized to be palmitoylated, though this was not confirmed (Frénal et al., 2010). While the palmitome purification methods used here are aimed at identifying palmitoylated proteins rather than specific modified cysteines, ABE can be used for this purpose. This is because cysteine residues that are not palmitoylated will be irreversibly bound by NEM before ABE, and are therefore not modified during sample processing. By contrast, palmitoylated cysteines are left with exposed thiol groups after ABE, and will be alkylated during sample processing by iodoacetamide treatment prior to gel electrophoresis. By examining PfGAP45 peptide spectra individually, we identified a specific cysteine (cys160) in the PfGAP45 C terminus that was not modified by NEM, but that had been carbamidomethylated by iodoacetamide (Figure 5E). This is direct evidence that GAP45 is palmitoylated at its C terminus and further validates the proposed PM-IMC linking function of GAP45 in *Toxoplasma* and *Plasmodium*. Further examination of peptide fragmentation spectra produced during the course of this work has allowed the identification of several other palmitoylation sites (PfCRT, at cys301, for example; Figure S4) but the large-scale analysis of specific palmitoylation sites will require extensive modification of the ABE protocol.

### The Dynamic Palmitome: 2-BMP Affects Palmitoylation on a Range of Proteins

**Figure 6:**
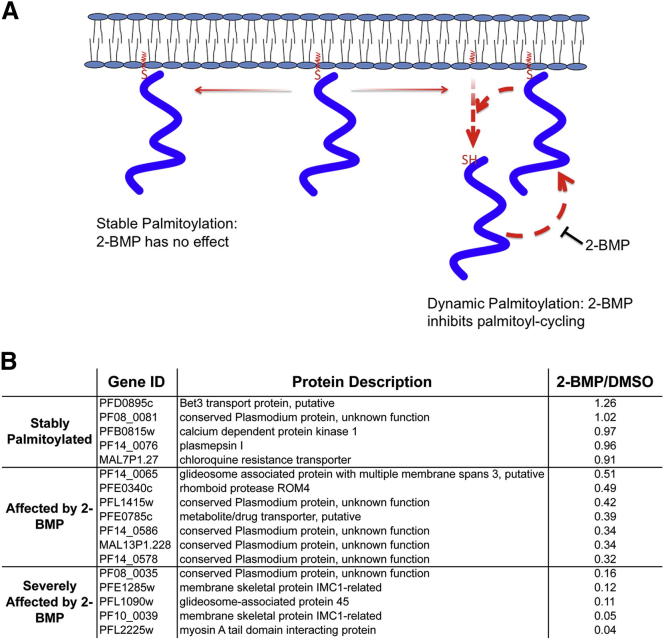
Palmitome Purification after 2-BMP Treatment Reveals Effects on *P. falciparum* Palmitoyl Proteins (A) A model illustrating 2-BMP’s potential effect on dynamically palmitoylated proteins. Proteins stably palmitoylated before 2-BMP addition will not be affected, while proteins subject to acyl cycling will be underrepresented due to inhibition of repalmitoylation. (B) A selected range of proteins identified by ABE from DMSO-treated *P. falciparum* schizonts are listed, with the effect of 2-BMP treatment determined by calculation of the abundance of these same proteins after ABE from 2-BMP-treated parasites and expressed as a ratio of 2-BMP/DMSO. Ratios less than 1 indicate protein loss after 2-BMP treatment. Full data sets from DMSO and 2-BMP treated parasites are in Tables S6 and S7. See also Figure S5 and Tables S6 and S7.

The destabilizing effect of 2-BMP on PfGAP45 and PfMTIP provides an explanation for its invasion-inhibitory effect; however, it is likely that 2-BMP also affects other palmitoyl proteins essential for invasion. To determine the range of proteins affected by 2-BMP treatment, and to provide a more complete explanation for its effect on schizont development and invasion, we used ABE to purify the palmitome from well-synchronized parasites treated for 6 hr with 50 μM 2-BMP or DMSO. To directly compare palmitoyl protein levels in 2-BMP versus DMSO samples, we used chemical isotope labeling to add an “intermediate” (DMSO) or “heavy” (2-BMP) isotope tag to peptides derived from the palmitome (+hydroxylamine) elutions of either sample (a “light” tag was used to label –hydroxylamine elutions and peptides were pooled prior to MS/MS analysis). Comparison of intermediate and heavy derived protein quantities then allowed the creation of 2-BMP/DMSO ratios describing the loss of specific palmitoyl proteins after 2-BMP treatment, with a ratio significantly less than 1 indicating protein loss (Figure 6B, Tables S6 and S7, and Figure S5A).

The 2-BMP/DMSO ratio of palmitoyl proteins purified after DMSO treatment indicate that 2-BMP has a significant impact on a number of palmitoyl proteins (Figure 6B and Table S6). It is important to note that the impact of 2-BMP treatment could have extended to a broader range of palmitoyl proteins with an increased 2-BMP exposure time; however, the short exposure time used here allows some insight into the range of palmitoylation-dependent regulatory mechanisms active in asexual-stage parasites. First, there are several proteins (PfBet3, PfCRT, PfCDPK1) that are unaffected by 2-BMP treatment. This could be an indication that these proteins are stably palmitoylated—that they were translated and palmitoylated prior to 2-BMP addition, and that once palmitoylated they were not depalmitoylated (Figure 6A). Second, there are several proteins that are reduced to an intermediate level after 2-BMP treatment (for example, PfGAPM3, PfEMP2, and PfROM4, with 2-BMP/DMSO ratios between 0.35 and 0.7). This intermediate level of palmitoyl protein loss could indicate an effect primarily on protein translated after drug addition, where protein translated before addition of 2-BMP is palmitoylated and protein translated after its addition is not. To illustrate, 2-BMP was added at approximately 38–42 hr after invasion, and it follows that the majority of palmitoyl proteins *affected* by 2-BMP would be maximally expressed at or after this time point in asexual-stage development. This hypothesis is supported by the expression data available for the DMSO and 2-BMP treated palmitomes, with the peak expression time for palmitoyl proteins affected by 2-BMP being skewed toward a later time point (Figure S5B). Lastly, the quantities of several proteins were severely reduced after 2-BMP treatment (2-BMP/DMSO ratios of 0.3 to 0.1). These include PfMTIP and PfGAP45, as well as several proteins that were not previously known to be palmitoylated. As we have shown with PfGAP45 and PfMTIP, the near complete loss of these proteins could be due to their destabilization, or it could be due to an inhibition of palmitoyl cycling (Figure 5A).

## Discussion

We have characterized the *P. falciparum* schizont-stage palmitome using two complementary palmitoyl protein purification techniques coupled with quantitative mass spectrometry. This has allowed the identification of more than 400 putative palmitoyl proteins in *P. falciparum*, many of which play central roles in asexual-stage development and virulence. This work provides valuable insight into the range of palmitoylation-regulated processes active in asexual-stage *P. falciparum*, but also raises a series of questions, relating first to the quality and depth of the palmitome as we have described it, second to the parasite’s means of palmitoylating the targets identified here, and lastly to the functional role played by palmitoylation in regulating these proteins.

Regarding the quality of the palmitome we have described, it must be noted that both ABE and MLCC will generate false positives for a range of reasons, as discussed above (Kang et al., 2008; Roth et al., 2006; Yang et al., 2010). However, by performing all experiments using SILAC we have been able to comprehensively define the *P. falciparum* palmitome based on quantitative enrichment data. This significantly increases the value of the data as a resource because the total enrichment data used for analysis is presented here and can be independently analyzed before beginning experimental follow-up on a particular protein (Table S5). It is also important to note that our cutoffs and groupings of enriched and highly enriched proteins have been completely unbiased regarding an expectation of palmitoylation. This is because any effort to eliminate proteins not expected to be palmitoylated would raise the likelihood that true positives are eliminated. For example, both ABE and MLCC identify several ribosomal proteins and histones, and while one might consider these false positives due to their abundance, it has recently been established in other systems that these proteins can be palmitoylated (Wilson et al., 2011; Yang et al., 2010).

While setting cutoff criteria is fundamentally arbitrary, it is possible to assess whether our criteria have created false negatives as a result of being too stringent. While no consensus site for palmitoylation has been defined, there is an easily identifiable consensus site for N-myristoylation, N-MGXXXS/T (Resh, 1999), and since N-myristoylation is often coupled to palmitoylation, it is possible to compare the number of predicted N-myristoyl-palmitoyl proteins present in the *P. falciparum* genome with the number that we have experimentally identified. This will also provide an indication of the depth of palmitome coverage. *P. falciparum* has 51 predicted N-myristoylated proteins (based on a search of soluble proteins for the above motif), 13 of which have a cysteine residue within 10 amino acids of the N-myristoylation motif, which is indicative of dual acylation. Ten of these 13 predicted dually acylated proteins are identified here, and eight are defined as palmitoyl proteins using our cutoff criteria (Figure S3). Of the three that are not identified, one is predominantly expressed in gametocytes, one is expressed at low levels in blood stages, and one is small and predicted to produce only one peptide identifiable by mass spectrometry after trypsin digestion. Having confirmed as palmitoylated eight out of ten predicted dually acylated proteins that are likely to be detectable suggests that we have identified the majority of palmitoyl proteins in *P. falciparum* schizonts.

Despite the depth of palmitome coverage reported here, palmitoyl proteins have likely been missed; however, this data set can be a useful guide for their identification. For example, we have identified the IMC components PfGAPM2 and PfGAPM3 as palmitoyl proteins but not the related protein PfGAPM1. PfGAPM1, like PfGAPM2 and PfGAPM3, does however have conserved cysteine residues within and adjacent to its TM domains (which are frequent sites of palmitoylation), making it a likely palmitoyl protein that we have either failed to detect or that is not palmitoylated at the time point we have sampled.

This data set confirms the widespread use of palmitoylation by *P. falciparum*, but how does the parasite regulate this modification? The distinguishing feature of palmitoylation in comparison with other acyl modifications is its reversibility, which is central to its role in protein regulation. The dynamic use of palmitoylation requires the ability to tightly control the addition and removal of palmitoyl groups, and the enzymology of palmitoylation is an active area of investigation. Work performed largely in model organisms or eukaryotic cell culture has identified two families of palmitoyl acyltransferases (PATs): the DHHC domain protein family that catalyze the palmitoylation of intracellular proteins, and the membrane-bound O-acyltransferase (MBOAT) protein family that catalyze the palmitoylation of secreted proteins. Additionally, two types of palmitoyl-thioesterase are responsible for protein depalmitoylation, the palmitoyl protein thioesterases (PPT1 and PPT2) and the acyl protein thioesterases (APT1 and APT2) (Resh, 2006a).

DHHC PATs are encoded in all eukaryotic genomes sequenced to date, with seven in the *S. cerevisiae* genome and 25 in the human genome (Mitchell et al., 2006). There are 12 DHHC-domain containing proteins encoded in the *P. falciparum* genome, and the expression profiles of these genes indicate expression of multiple members at each life-cycle stage. One *P. falciparum* DHHC-protein has been characterized, PFC0160w, and it is targeted to the Golgi (Seydel et al., 2005). As palmitoylation of a given protein depends on it having access to a DHHC-PAT, the localization of DHHC proteins may impart a basic level of palmitoyl protein regulation. The *P. falciparum* genome encodes one MBOAT family homolog, which is expressed throughout the life cycle. Previous work suggests this protein is essential (Palacpac et al., 2004), and given that we have identified several exported palmitoyl proteins, it will be important to determine whether this protein is responsible for palmitoylating the secreted proteins identified here. Lastly, PPT and APT family palmitoyl-thioesterases are not well characterized and their overall sequence conservation is low; however, specific structural domains are known to be functionally important, and *P. falciparum* encodes several proteins with the necessary alpha/beta hydrolase fold, though the precise number of *P. falciparum* PPT or APT homologs is unclear (Cantu et al., 2010).

The *P. falciparum* genome clearly encodes the enzymes necessary for the tight control of palmitoylation, but how might this PTM be used to regulate specific *P. falciparum* proteins? In the case of TM domain proteins, palmitoylation might be used to regulate within-membrane localization or protein stability. For example, the trafficking, within-membrane localization, membrane retention, and gating of many multipass membrane channels is influenced by palmitoylation (Shipston, 2011), and we have identified a wide range of palmitoylated *P. falciparum* membrane channels here (PfCRT, for example), which could be regulated in a similar manner. Palmitoylation can also affect single-pass TM proteins, such as the yeast SNARE, Tlg1, which is degraded by the proteasome if it is not palmitoylated (Valdez-Taubas and Pelham, 2005). Furthermore, the majority of raft-associated TM proteins are palmitoylated, with this being important for their within-membrane sorting (Levental et al., 2010), and palmitoylation of single-pass TM proteins may play a similar role in *P. falciparum*.

With soluble palmitoyl proteins, palmitoylation likely regulates membrane binding and associated processes. More than 50% of the palmitoyl protiens we have identified are soluble. These include structural (alveolins), signaling (kinases and phosphatases), metabolic (glycolytic enzymes), chaperone (HSPs), and hypothetical proteins, providing insight into the diversity of palmitoylation-regulated processes in *P. falciparum*. Regulation of these palmitoylation targets could involve cyclic acylation, like H/NRas (Rocks et al., 2005), or they could be targets of “palmitoyl switches,” with a palmitoyl moiety being added or revealed after phosphorylation or ligand biding (Salaun et al., 2010). The identification of a second palmitoylation site in PfGAP45 is, in this instance, interesting because it is adjacent to two phosphorylation sites (Ser156 and Thr158 [Treeck et al., 2011]), providing indirect support for a hypothesized relationship between these two PTMs (Jones et al., 2009).

In summary, we have used a multitiered approach to identify a significant portion of the *P. falciparum* palmitome and have shown that palmitoylation may play a role in multiple parasite-specific processes of great biological interest. This data should provide a valuable resource for the field and allow specific hypotheses to be generated for individual proteins of interest, particularly for those with no known function.

## Experimental Procedures

### *P. falciparum* Culture, SILAC Labeling, and Transfection

*P. falciparum* strain 3D7 was cultured in O^+^ human erythrocytes and 10% human serum or 0.5% Albumax I (Invitrogen) in RPMI-based media as described (Trager and Jensen, 1976). Use of erythrocytes and serum from human donors for parasite culture was approved by the NHS Cambridgeshire 4 Research Ethics Committee, and all donors supplied written informed consent. SILAC labeling was performed as described (Nirmalan et al., 2004). Transfection was performed as described (Nkrumah et al., 2006).

### *P. falciparum* Palmitome Purification

Both ABE and MLCC were performed as described (Martin and Cravatt, 2009; Wan et al., 2007). For palmitome purification from 2-BMP or DMSO treated parasites, cultures were treated with 50 μM 2-BMP (2-bromohexadecanoic acid, Sigma) or DMSO for 6 hr before collection for ABE. For western blotting of palmitome samples, aliquots of each were separated by SDS-PAGE, transferred to nitrocellulose, and probed with specific antisera as described (Jones et al., 2006).

### Mass Spectrometry and Data Analysis

For analysis of SILAC ABE and MLCC samples, control (light) and palmitome (heavy) eluates were pooled and separated by SDS PAGE. Gels were stained with colloidal Coomassie (Sigma) and bands were excised, destained, and proteins were in-gel digested. Peptides were analyzed by LC-MS/MS with an LTQ Orbitrap Velos mass spectrometer (Thermo). For palmitome analysis after 2-BMP or DMSO treatment, samples were separated by SDS-PAGE and processed as above. Peptides from 2-BMP or DMSO samples were dimethyl stable isotope-labeled as described (Boersema et al., 2009) and pooled for LC-MS/MS analysis. MS data files were converted to PRIDE XML files with PRIDE Converter v2.5.0 and uploaded to the PRIDE database (http://www.ebi.ac.uk/pride/) with accession numbers 17888–17897.

Data from SILAC experiments was analyzed with MaxQuant version 1.0.13.13 and Mascot server 2.2 (Matrix Science) (Cox and Mann, 2008) and dimethyl labeled peptide data was analyzed with MaxQuant version 1.1.1.36 with the integrated search engine Andromeda (Cox et al., 2011). MaxQuant processed data was searched against a combined Human (IPI) and Plasmodium falciparum (GeneDB) database. A protein FDR of 0.01 and a peptide FDR of 0.01 were used for identification level cutoffs. Cutoff criteria for SILAC experiments were decided individually for each purification method and for the ABE/MLCC overlap. Criteria were based on both individual ratio count and individual enrichment ratios in comparison to aggregate ratios. Criteria for palmitome analysis after DMSO or 2-BMP treatment were based on individual enrichment ratios in comparison to aggregate ratios. Statistical analysis of SILAC experiments was performed with Perseus (a MaxQuant statistical package) considering only proteins with ratios in at least three of five SILAC experiments. Significantly enriched proteins were identified by t testing with Benjamini and Hochberg adjustment to generate a false discovery rate of 5%.

### Analysis of 2-BMP-Treated Parasites and PfGAP45 Mutational Analysis

For analysis of 2-BMP-treated *P. falciparum* by electron microscopy, parasites were treated for 24 hr with 100 μM 2-BMP or DMSO before being collected and processed as described (Moon et al., 2009). For determination of the effect of 2-BMP on invasion, schizonts were treated with 2-BMP or DMSO, and rings were counted by flow cytometry as described (Theron et al., 2010). To determine whether the effect of 2-BMP was on schizonts or erythrocytes, both were separately treated with 2-BMP or DMSO, washed, mixed, and invasion was measured as described (Theron et al., 2010). For all assays, invasion in DMSO controls was considered 100% and the effect of 2-BMP was compared to DMSO to determine invasion efficiency.

To determine the effect of 2-BMP on the glideosome, schizonts were treated for 4 hr with 50 μM 2-BMP, 50 μM 2-BMP and 20 μM MG-132 (Calbiochem), or DMSO before collection for western blotting and IFA analysis, performed as described (Jones et al., 2006; Tonkin et al., 2004). For mutational analysis of PfGAP45, PfGAP45-HA and PfGAP45-Npal-HA lines were created using the Bxb1 integrase system with the 3D7attB parasite line (Nkrumah et al., 2006). Western blotting and IFA of transfected lines was performed as above. Densitometry was performed with ImageJ (available at http://rsbweb.nih.gov/ij/).

### Supplemental Experimental Procedures

For complete details of all experiments and analysis methods, see the Supplemental Experimental Procedures.

## Figures and Tables

**Table 1:** *P. falciparum* Palmitoyl Proteins Identified by Both ABE and MLCC

Gene ID	Protein Description	Mean ABE Ratio	Mean CC Ratio
**TM Domain Proteins**			

PF14_0541	V-type H(+)-translocating pyrophosphatase, putative	3.60	2.43
PFB0210c	hexose transporter, PfHT1	4.08	2.58
PF11_0172	folate/biopterin transporter, putative	3.32	3.51
PF14_0679	inorganic anion exchanger, inorganic anion antiporter	5.96	3.77
PFI0720w	transporter, putative	3.79	3.09
MAL7P1.27	chloroquine resistance transporter	13.54	6.65
PFE0785c	metabolite/drug transporter, putative	6.12	3.55
PFL1125w	phospholipid-transporting ATPase, putative	6.39	2.11
PF13_0252	nucleoside transporter 1	5.58	3.36
MAL13P1.231	Sec61 alpha subunit, PfSec61	3.28	3.31
PFE1130w	conserved protein, unknown function	5.80	3.35
PFF1375c	ethanolaminephosphotransferase, putative	3.16	3.32
PF14_0528	hemolysin, putative	3.99	3.38
PF14_0607	conserved *Plasmodium* membrane protein, unknown function	5.55	6.03
MAL13P1.329	conserved *Plasmodium* membrane protein, unknown function	3.88	2.26
PF11_0338	aquaglyceroporin	3.83	2.42
PF14_0065	conserved *Plasmodium* membrane protein, unknown function	9.93	7.77
PFC0725c	formate-nitrate transporter, putative	4.06	2.74
PFE0340c	rhomboid protease ROM4	8.92	5.01
MAL13P1.298	conserved *Plasmodium* membrane protein, unknown function	3.34	8.77
PF10_0366	ADP/ATP transporter on adenylate translocase	3.95	2.37
PF13_0272	thioredoxin-related protein, putative	3.79	3.11
PF14_0493	sortilin, putative	3.57	2.07
MAL13P1.56	m1 family aminopeptidase	3.09	2.61
PF11_0052	Qa-SNARE protein, putative	9.71	7.47
PF11_0055	conserved protein, unknown function	3.09	3.57
PF14_0567	conserved *Plasmodium* protein, unknown function	3.32	2.14
PFE1590w	early transcribed membrane protein 5, ETRAMP5	3.09	2.54
PFF1415c	DNAJ domain protein, putative	4.01	4.42
PFL1415w	conserved *Plasmodium* protein, unknown function	8.46	3.90

**Dual Acylation Motif or Cysteine Near the N or C terminus**			

PF10_0039	membrane skeletal protein IMC1-related	4.27	9.57
PF10_0107	conserved protein, unknown function	16.78	8.46
PF11_0211	conserved *Plasmodium* protein, unknown function	8.06	4.36
PF11_0415	conserved *Plasmodium* protein, unknown function	3.64	10.77
PF14_0578	conserved *Plasmodium* protein, unknown function	9.41	9.97
PF14_0586	conserved *Plasmodium* protein, unknown function	7.08	6.61
PFB0815w	calcium-dependent protein kinase 1	4.71	2.53
PFE0660c	purine nucleotide phosphorylase, putative	4.30	2.74
PFE1285w	membrane skeletal protein IMC1 related	5.63	8.91
PFF0380w	conserved *Plasmodium* protein, unknown function	7.92	4.38
PFL1090w	glideosome-associated protein 45	9.74	11.53
PFL2225w	myosin A tail domain-interacting protein	4.01	8.26

**Atypical**			

PF07_0127	conserved *Plasmodium* protein, unknown function	9.86	5.01
PF08_0035	conserved *Plasmodium* protein, unknown function	4.11	5.23
PF10_0126	conserved *Plasmodium* protein, unknown function	9.14	4.98
PF10_0220	phospholipid scramblase 1, putative	10.65	12.64
PF11_0164	peptidyl-prolyl cis-trans isomerase	3.87	2.92
PF13_0322	falcilysin	3.42	2.26
PFC0120w	cytoadherence-linked asexual protein 3.1	3.23	2.65
PFC0170c	dihydrolipoamide acyltransferase, putative	3.65	2.38
PFC0745c	proteasome component C8, putative	4.47	2.69
PFE0040c	mature parasite-infected erythrocyte surface antigen (MESA) or PfEMP2	8.43	7.26
PFF0675c	myosin E	6.01	3.30
PFI0175w-a	conserved *Plasmodium* protein, unknown function	5.25	4.77
PFI0265c	RhopH3	4.35	2.65

A selected set of 55 proteins are listed and divided into groups that represent common palmitoyl protein types or that do not represent a common palmitoyl protein class.

## Supplemental Information

Table S1. Proteins Enriched and Highly Enriched by Acyl-biotin Exchange, Related to Figure 2 Proteins also enriched or highly enriched by metabolic labeling and click chemistry (Table S2) are highlighted in red.

Table S2. Proteins Enriched and Highly Enriched by Metabolic Labeling and Click Chemistry, Related to Figure 2 Proteins also enriched or highly enriched by ABE (Table S1) are highlighted in red.

Table S3. Total Proteins Enriched by Acyl-Biotin Exchange and Metabolic Labeling and Cick Chemistry and the Overlap between These Enriched Proteins, Related to Figure 3 Total complement of proteins enriched by acyl-biotin exchange and metabolic labeling and click chemistry without consideration of ratio count (sheets 1 and 2) and the overlap between these two data sets (sheet 3).

Table S4. The Total *P. falciparum* Palmitome, Related to Figure 3 Proteins listed here are the total nonredundant proteins listed in Tables S1, S2, and S3 and are the total complement of proteins with experimental evidence of enrichment after palmitoyl protein purification. All proteins identified as significantly enriched after statistical analysis with Perseus (p values listed in last column; 5% false discovery rate) are highlighted in blue.

Table S5. Total MaxQuant Results for All Biological Replicates, Related to Figure 3 This table also includes all available percent ^13^C_6_,^15^N_1_-isoleucine incorporation data for each protein.

Table S6. Protein Enrichment by Acyl-Biotin Exchange after DMSO or 2-BMP Treatment, Related to Figure 6 Proteins enriched after acyl-biotin exchange from DMSO- (sheet 1) or 2-BMP- (sheet 2) treated parasites and proteins less enriched after 2-BMP treatment (sheet 3). In sheet 3, proteins that are enriched by *both* ABE and MLCC are highlighted in red.

Table S7. Total MaxQuant Results for Palmitome Purification from DMSO- or 2-BMP-Treated Parasites, Related to Figure 6

Document S1. Supplemental Experimental Procedures and Figures S1–S5
