# Supplementary material for: Analysis of Protein Palmitoylation Reveals a Pervasive Role in Plasmodium Development and Pathogenesis
Source: Cell Host Microbe. 2012 Aug 16;12(2):246–58. doi: 10.1016/j.chom.2012.06.005 (PMC3501726; doi:10.1016/j.chom.2012.06.005)
Supplement: Document S1. Supplemental Experimental Procedures and Figures S1–S5 [file mmc8.pdf]

Cell Host & Microbe, *Volume 12*

Supplemental Information

Analysis of Protein Palmitoylation Reveals a Pervasive Role in *Plasmodium* Development and Pathogenesis

Matthew L. Jones, Mark O. Collins, David Goulding, Jyoti S. Choudhary, and Julian C. Rayner

Supplemental Experimental Procedures

P. falciparum SILAC labeling

For SILAC labeling, *P. falciparum* strain 3D7 was cultured in 2.5-5% O^+ human erythrocytes with 0.5% Albumax I in custom-made RPMI media lacking isoleucine (Invitrogen) and supplemented with "light" or "heavy" isoleucine at 60mg/L. For all experiments, *P. falciparum* cultures were grown overnight in the absence of isoleucine before being switched to custom-RPMI with standard (light) isoleucine or isotopic (heavy) isoleucine ($^{13}C_6$,$^{15}N_1$-isoleucine; Cambridge Isotope Laboratories) to increase the mass of heavy isoleucine-containing peptides by 7Da. In order to ensure maximum incorporation of isotopic isoleucine and to generate enough material for substantial purification of palmitoyl-proteins, all cultures were maintained for three generations in light or heavy media before collection.

P. falciparum palmitome purification by acyl-biotin exchange

For the large-scale purification of palmitoyl-proteins by ABE, *P. falciparum* cultures were SILAC labeled as described above and parasites were collected by saponin lysis. Briefly, infected erythrocytes were pelleted and resuspended in 5-10mls of 0.1% Saponin lysis buffer (0.1% Saponin in PBS) and incubated at room temperature for 5min before being centrifuged at 3200 x G for 10min. After centrifugation, the supernatant was discarded and the parasite pellet was washed with 0.1% Saponin lysis buffer and centrifuged again at 3200 x G for 10min. Washes with 0.1% Saponin lysis buffer were repeated until supernatants were completely clear.

Saponin-treated parasite pellets were subsequently used for ABE. First, pellets were washed in ice-cold LB buffer (Lysis buffer: 50mM Tris, 150mM NaCl, 5mM EDTA, pH 7.4) and then resuspended at 1 x 10^9 parasites/ml in ice-cold LB buffer with 1.7% TX-100 and 10mM N-ethylmaleamide (NEM) with protease (Protease inhibitor cocktail, Sigma) and phosphatase inhibitors (Phosphatase inhibitor cocktail 2, Calbiochem) and incubated at 4°C for 1hr with rotation. Parasite lysates where then chloroform/methanol (C/M) precipitated (Wessel and Flugge, 1984) and precipitated protein was resuspended in 4SB (Solubilization buffer: 4% SDS, 50mM Tris, 5mM EDTA, pH7.4) with 10mM NEM and placed at 37°C with shaking until protein was fully solubilized. Solubilized protein was then diluted in LB buffer with 1mM NEM, 0.2% TX-100 and protease and phosphatase inhibitors and placed at 4°C overnight with rotation. After NEM treatment,

samples were subjected to three sequential C/M precipitations to fully remove NEM (resulting precipitated protein pellets were resolubilized with 4SB and then diluted with LB before being precipitated again). After final C/M precipitation, pellets were resolubilized in 4SB and like samples were pooled and protein concentrations were measured using the BCA test (Sigma; QPBCA-1KT). Equal amounts of protein from light SILAC-labeled cultures and heavy SILC-labeled cultures were then either mock-treated with hydroxylamine (light) or treated with hydroxylamine (heavy) to cleave thioester bonds. For mock treatment, protein solubilized in 4SB from light SILAC cultures was diluted with –hydroxylamine buffer (50mM Tris, 1mM HPDP-biotin, 0.2%TX100, pH7.4) and incubated at room temperature for 2hrs with rotation. For hydroxylamine treatment, protein solubilized in 4SB from heavy SILAC cultures was diluted with +hydroxylamine buffer (0.7M Hydroxylamine, 1mM HPDP-biotin, 0.2% TX100, pH 7.4) and incubated at room temperature for 2hrs with rotation. After (-) or (+) hydroxylamine treatment, samples were C/M precipitated and further treated with HPDP-biotin by first resolubilizing pellets in 4SB and then adding low-HPDP-biotin buffer (50mM Tris, 150mM NaCl, 5mM EDTA, 0.2mM HPDP-biotin, 0.2% TX100). Treatment with low-HPDP-biotin buffer was for 2hrs at room temperature with rotation. Excess HPDP-biotin was removed by three sequential C/M precipitations. After the final C/M precipitation, pellets were resuspended in 2SB (2% SDS, 50mM Tris, 5mM EDTA, pH7.4) and then SDS was diluted to 0.1% by addition of LB buffer with 0.2% TX-100 and protease and phosphatase inhibitors. Samples were centrifuged at 13000 x G briefly to remove particulates and 15μl/ml sample of streptavidin-agarose (Thermo) was added. Samples were incubated with streptavidin-agarose for 1.5-2hrs at room temperature with rotation. Streptavidin-agarose beads were then precipitated by centrifugation at 3200 x G and washed 4 times with LB buffer with 0.1% SDS and 0.2%TX-100 before elution by incubation at 37°C with agitation in 2-3 bead volumes LB buffer with 0.1% SDS, 0.2% TX-100 and 1% β-mercaptoethanol.

P. falciparum palmitome purification by metabolic labeling/click chemistry

For the large-scale purification of *P. falciparum* palmitoyl-proteins by metabolic labeling/click chemistry, cultures were SILAC labeled as described above and concurrently labeled with the palmitic acid analogue, 17-octodecynoic acid (Cayman Chemical), or grown with an equal volume of DMSO. Metabolic labeling with 17-ODYA was at 25μM for 24hrs (from late rings/early trophozoites to mid-late schizonts). 17-ODYA labeled and mock labeled parasites were collected by saponin lysis as described above.

To isolate 17-ODYA labeled proteins, saponin pellets from 17-ODYA labeled "heavy" and DMSO "light" parasites were solubilized in 2% SDS in PBS (pellets were resuspended at 5×10^8-1×10^9 parasites/ml) with protease and phosphatase inhibitors and heated at 95°C for 5 min before centrifugation for 5min at 18000 x G. Resulting supernatants were C/M precipitated to remove excess 17-ODYA and precipitated protein was resuspended again in 2% SDS/PBS. Protein concentrations from 17-ODYA or DMSO labeled parasites were then measured using a BCA test and equal protein quantities were subjected to click chemistry to biotinylate 17-ODYA-labeled proteins. For click chemistry, 1-2mg/ml of protein was added to 100μM Biotin-azide

(Invitrogen), 1mM Tris(2-carboxyethyl)phosphine (TCEP, Sigma), 100μM Tris[(1-benzyl-1H-1,2,3-triazol-4-yl)methyl]amine (TBTA, Sigma) dissolved in DMSO/*tert*-butanol (20%/80%), and 1mM $CuSO_4$ (Sigma) in a reaction made up to 1ml with PBS. The click chemistry reaction proceeded for 1-1.5hrs before being stopped by C/M precipitation. After resuspension in 2% SDS/PBS, C/M precipitation was repeated to ensure complete removal of biotin-azide. For purification of biotinylated proteins, precipitated protein resuspended in 2% SDS/PBS was diluted 10X with 0.2% TX-100/PBS and streptavidin-agarose (10μl/ml sample) was added. Samples were then incubated with rotation at room temperature for 2hrs or overnight at 4°C. Streptavidin-agarose was collected by centrifugation at 3200 x G for 5min and agarose beads were washed 4X with 10mls 0.2% TX-100/PBS. Elution of precipitated proteins was performed by heating agarose beads at 95°C for 5min in double the bead volume 2% SDS in 50mM Tris, 5mM EDTA, pH 7.4.

To determine whether 17-ODYA is linked to proteins via a thioester bond, parasites were labeled with 17-ODYA and subjected to click-chemistry as described above. Biotinylated proteins were captured as described above, but before elution, streptavidin-agarose beads were treated with 1M hydroxylamine pH 7.4 or PBS for 1 hour to cleave thioester bonds. Elution of biotinylated proteins then proceeded as described above and eluates were probed by Western blotting for the presence of PfGAP45 and PfCRT.

Palmitome purification from 2-BMP treated *P. falciparum*

ABE with 2-BMP or DMSO treated parasite material was performed as described above with (+) or (-) hydroxylamine treatment being performed on material exclusively derived from 2-BMP or DMSO treated parasites. Resulting ABE palmitome and control elutions (one palmitome and control elution set from 2-BMP treated parasites and one palmitome and control elution set from DMSO treated parasites) were separated by 1D-SDS PAGE and subjected to quantitative MS/MS analysis as described below.

Quantitative Mass Spectrometry

For analysis of SILAC ABE and metabolic labeling/click chemistry eluates, equal volumes of control (light) and palmitome (heavy) eluates were pooled and concentrated by centrifugation on a VIVASPIN filtration column (3000 kDa molecular weight cut-off; Sartorius Stedim Biotech). Pooled and concentrated eluates were reduced, alkylated, and separated by 1-D SDS PAGE. For analysis of $^{13}C_6$,$^{15}N_1$-isoleucine (heavy) incorporation, aliquots of the ABE and metabolic labeling/click chemistry "heavy" elutions were reduced, alkylated, and separated individually by 1-D SDS PAGE. Protein gels were stained overnight with colloidal Coomassie blue (Sigma). Each lane was excised into 12 bands that were destained and in-gel digested overnight using trypsin (sequencing grade; Roche). Peptides were extracted from gel bands twice with 50% acetonitrile/0.5% formic acid and dried in a SpeedVac (Thermo) and were resuspended using 0.5% formic acid prior to LC-MS/MS analysis.

For the quantitative analysis of 2-BMP or DMSO treated ABE palmitome and control sets, samples were run separately on SDS PAGE gels, stained overnight with colloidal Coomassie blue and gel lanes were excised into 12 gel sections. Extracted peptides from equivalent gel bands were

labeled with dimethyl stable isotopes (N-terminal and lysine residues) essentially as described (Boersema et al., 2009) using a combination of formaldehyde and sodium cyanoborohydride for light labeled samples (28 Da mass addition), D2 formaldehyde and sodium cyanoborohydride for medium/intermediate labeled samples (32 Da mass addition) and 13CD2 formaldehyde and sodium cyanoborodeuteride for heavy labeled samples labeled (36 Da mass addition). Peptides from control elutions were pooled and chemically labeled "light", peptides from the (+) hydroxylamine elutions from DMSO treated parasites were chemically labeled "intermediate" and peptides from (+) hydroxylamine elutions from 2-BMP treated parasites were chemically labeled "heavy". Labeled peptides were pooled for analysis by LC-MS/MS.

Peptide samples from each gel band were analysed online using an Ultimate 3000 Nano/Capillary LC System (Dionex) coupled to an LTQ Orbitrap Velos hybrid mass spectrometer (Thermo Electron) equipped with a nanospray ion source. Peptides were desalted on-line using a micro-Precolumn cartridge (C18 Pepmap 100, LC Packings) and then separated using a 60 min RP gradient (4-32% acetonitrile/0.1% formic acid) on a BEH C18 analytical column (1.7um, 75 µm id x 10 cm,) (Waters). The mass spectrometer was operated in standard data dependent acquisition mode controlled by Xcalibur 2.1. The instrument was operated with a cycle of one MS (in the Orbitrap) acquired at a resolution of 60,000 at m/z 400, with the top 10 most abundant multiply-charged (2+ and higher) ions in a given chromatographic window subjected to MS/MS fragmentation in the linear ion trap. An FTMS target value of 1e6 and an ion trap MSn target value of 5000 were used. The maximum FTMS scan accumulation time was set at 250ms and maximum ion trap MSn scan accumulation time was set at 30ms. Dynamic exclusion was enabled with a repeat duration of 45s with an exclusion list of 500 and exclusion duration of 30s. Acquisition was performed for 65 min for each gel band. MS data files were converted to PRIDE XML files using PRIDE Converter v2.5.0 and uploaded to the PRIDE database (http://www.ebi.ac.uk/pride/) with accession numbers 17888-17897.

Data Analysis

Data from SILAC experiments was analysed using MaxQuant version 1.0.13.13 and Mascot server 2.2 (Matrix Science) (Cox and Mann, 2008) and dimethyl labeled peptide data was analysed using MaxQuant version 1.1.1.36 with the integrated search engine Andromeda (Cox et al., 2011). MaxQuant processed data was searched against a combined Human (IPI) and Plasmodium falciparum (GeneDB) sequence database using the following search parameters: trypsin with a maximum of 2 missed cleavages, 7 ppm for MS mass tolerance, 0.5 Da for MS/MS mass tolerance, with Acetyl (Protein N-term), Oxidation (M), Deamidated (NQ), carbamidomethyl (C) and Nethylmaleimide (C) set as variable modifications. A protein FDR of 0.01 and a peptide FDR of 0.01 were used for identification level cut offs. Protein quantification was performed using razor and unique peptides.

To individually group proteins identified by ABE or metabolic labeling/click chemistry into enriched or highly enriched classes, we have used the definitions and cut-off criteria outlined below.

Definitions:

- *Mean Ratio:* mean of MaxQuant-generated ratios (palmitome (heavy)/control (light)) from each biological replicate for each individual protein identified
- *Median or Mean aggregate ratio:* the median or mean of the individual mean ratios for each group of proteins being considered
- *Median or Mean Absolute Deviation:* the median or mean of the absolute deviation of each individual protein's ratio from the median or mean aggregate ratio
- *Max mean ratio:* the greater of the mean ratios generated for an individual protein before and after normalizing enrichment ratios to account for less than 100% incorporation of $^{13}C_6$,$^{15}N_1$-isoleucine

For ABE:

1. *Enriched Proteins:* all proteins identified by a cumulative total of 4 or more peptides across 3 biological replicates and with a mean ratio greater than the median aggregate ratio plus the median absolute deviation. Proteins were also considered enriched if they met the above peptide count criteria and their max mean ratio was greater than the median aggregate ratio plus median absolute deviation calculated after normalization to account for percent $^{13}C_6$,$^{15}N_1$-isoleucine incorporation.
2. *Highly Enriched Proteins:* all proteins identified by a cumulative total of 5 peptides in 2 of 3 biological replicates and with a mean ratio greater than the mean aggregate ratio plus the mean absolute deviation

For Metabolic labeling/click chemistry:

1. *Enriched Proteins:* all proteins identified by a cumulative total of 3 or more peptides across 2 biological replicates and with a mean ratio greater than the median aggregate ratio. Proteins were also considered enriched if they met the above peptide count criteria and their max mean ratio was greater than the median aggregate ratio calculated after normalizing to account for percent $^{13}C_6$,$^{15}N_1$-isoleucine incoroporation.
2. *Highly Enriched Proteins:* all proteins identified by a cumulative total of 4 peptides across 2 replicates and with a mean ratio greater than the mean aggregate ratio

To group proteins identified by ABE and metabolic labeling/click chemistry into an "overlap" group (Figure 3), we first used less stringent criteria to create more inclusive lists of proteins classified as enriched by *either* ABE *or* metabolic labeling/click chemistry. We next compiled the proteins identified as enriched by *both* palmitome purification methods with these relaxed criteria into the "overlap" group. This group was created with the definitions and cut-off criteria outlined below.

Definitions:

- *Minimum and maximum ratios:* the lowest and highest individual MaxQuant-generated ratios for an individual protein across biological replicates.
- *Median minimum ratio:* the median of all minimum ratios for the total set of proteins identified by either ABE or metabolic labeling/click chemistry

- *Median absolute deviation (minimum):* the median of the absolute deviation of each individual protein's minimum ratio from the median minimum ratio

For creation of the Overlap group:

1. *ABE enriched:* all proteins with a maximum ratio greater than the median minimum ratio plus the median absolute deviation (minimum). Peptide counts are not considered
2. *Metabolic labeling/click chemistry:* all proteins with a maximum ratio greater than the median minimum ratio. Peptide counts are not considered
3. *Overlap:* all proteins common to 1 and 2

Statistical Analysis of Enrichment (Perseus)

Statistical analysis of the entire dataset was performed using Perseus, a statistical package associated with MaxQuant. Data was filtered so that proteins were only included if ratios were available for at least 3 out of 5 replicate ABE and MLCC experiments. Protein ratios were Log2 transformed, normalized to the median and significantly enriched proteins were identified by t-testing with Benjamini and Hochberg adjustment to generate a false discovery rate of 5%.

The ABE-purified palmitome from DMSO or 2-BMP treated *P. falciparum*

To distinguish palmitoyl-proteins from background in ABE-purified palmitome samples taken from DMSO or 2-BMP treated parasites, we used similar definitions and cut-off criteria to those outlined above for identification of proteins enriched by ABE. In brief, we considered all proteins from DMSO-derived palmitome (+hydroxylamine) samples enriched if their enrichment ratios (DMSO-palmitome (intermediate)/Control (light)) were greater than the median ratio plus the median absolute deviation. The same criteria were used to determine palmitoyl-protein enrichment in 2-BMP-palmitome samples: enrichment ratios (2-BMP-palmitome (heavy)/control (light)) greater than the median ratio plus the median absolute deviation were considered enriched. To determine which palmitoyl-proteins were significantly affected by 2-BMP treatment, we considered all proteins with a 2-BMP/DMSO ratio (heavy/intermediate) *less* than the median ratio *minus* the median absolute deviation to be significantly affected. Expression profiles of all palmitoyl-proteins identified in the DMSO or 2-BMP palmitome were obtained from PlasmoDB (plasmodb.org; GS-microarray expression profiles deposited by the Derisi Lab; (Bozdech et al., 2003)).

Mutational analysis of PfGAP45 N-terminal palmitoylation

Transfection of mutant and wild-type *PfGAP45* was achieved using the Bxb1 integrase system with the 3D7attB parasite line (Nkrumah et al., 2006). To properly express mutant and wild-type *PfGAP45*, the vector, pLN-ENR-GFP (Nkrumah et al., 2006), was modified to allow expression of *PfGAP45* under the control of the *PfAMA1* promoter with an HA-epitope tag. Transfection was performed as follows: 100-150ug of both pINT (Nkrumah et al., 2006) and the attP-containing PfGAP45 vector(s) were mixed and electroporated (0.31Kv and 950µF) into uninfected human erythrocytes. 3D7-attB parasites, cultured continuously in 2.5nM WR99210, were added to these plasmid-loaded erythrocytes and cultured for 48hrs before the addition of 2.5ug/ml BSD (Blastacidin-S deaminase) and 100ug/ml G418. Drug-resistant

parasites appeared between 16 and 21 days post-transfection, at which point G418 selection was removed to allow loss of pINT. Integration of the attP-containing PfGAP45 vector(s) was confirmed by PCR as previously described (Nkrumah et al., 2006).

For Western blotting of transfected parasite lines, PfGAP45-HA or PfGAP45-Npal-HA expressing schizont infected erythrocytes were lysed with 0.1% Saponin in PBS as described above and schizonts were solubilized in 1X Lamelli Sample Buffer with 5% β-mercaptoethanol. Western blotting of resulting lysates was performed as described in main experimental procedures. Immunofluorescence (IFA) of transfected parasites was performed as follows. Briefly, to enrich for late schizonts and merozoites, 10uM E-64 was added to mid-schizont stage cultures (40-42 hrs post-invasion) that were maintained for a further 6-8 hrs before purification of schizonts/merozoites by centrifugation over a 45% Percoll cushion. Purified late schizonts/merozoites were resuspended in a small volume of warm RPMI and dotted onto glass slides and allowed to air dry. Slides were submerged in ice-cold methanol for 5 min, allowed to air dry, and blocked with 3% BSA in PBS. Slides were stained with anti-HA or anti-PfGAP45 primary antisera and the appropriate secondary antisera before being visualized using an Olympus BX60 fluorescence microscope with a Diagnostic Instruments Spot RT monochrome camera. Images were merged using Adobe Photoshop 5.0.

Analysis of 2-BMP treated parasites

For analysis of 2-BMP treated parasites by electron microscopy, *P. falciparum* cultures were incubated overnight in either 100µM 2-BMP (2-bromohexadecanoic acid, Sigma) or an equivalent volume of DMSO before being collected and fixed firstly in 2% PFA with 2.5% GA in 0.1M sodium cacodylate buffer at pH7.42 with added 0.1% and 0.05% magnesium and calcium chloride at 20°C for 15 minutes and then on ice for the remainder of 1 hour. Secondary fixation was with 1% osmium tetroxide in sodium cacodylate buffer only at room temperature for 1 hour. The samples were mordanted with 1% tannic acid for 30 minutes and dehydrated through an ethanol series, staining en bloc with 2% uranyl acetate at the 30% stage before embedding in Agar 100 resin. 50nm ultrathin sections cut on a UCT ultramicrotome were collected onto copper grids, contrasted with uranyl acetate and lead citrate, viewed on an FEI 120kV Spirit Biotwin TEM, and imaged with an F4.15 Tietz CCD camera.

To determine the effect of 2-BMP treatment on erythrocyte invasion, late schizonts were incubated with increasing amounts of 2-BMP or DMSO for 12-16 hrs and rings were counted by flow cytometry as previously described (Theron et al., 2010). Invasion by DMSO treated parasites was considered 100% and the effect of 2-BMP treatment was measured against corresponding DMSO controls to determine invasion efficiency. To determine whether the effect of 2-BMP was on *P. falciparum* schizonts or on the erythrocytes being invaded, a two-color flow-cytometry based assay was used as previously described (Theron et al., 2010). Briefly, target erythrocytes were labeled with the cell dye, DDAO-SE (Invitrogen), and treated for 4 hours with DMSO or increasing concentrations of 2-BMP. *P. falciparum* schizont cultures were concurrently treated with either DMSO or increasing concentrations of 2-BMP. All treated cells were washed extensively and 2-BMP treated schizonts

were mixed with labeled DMSO-treated erythrocytes while DMSO-treated schizonts were mixed with labeled 2-BMP-treated erythrocytes. After a 12-16hr incubation, invasion into labeled erthrocytes was counted by flow-cytometry as previously described (Theron et al., 2010). Invasion in DMSO controls was considered 100% and the effect of 2-BMP treatment was measured against corresponding DMSO controls to determine invasion efficiency.

To determine the effect of 2-BMP treatment on the *P. falciparum* schizont glideosome, late blood-stage parasites were treated for 4 hours with 50μM 2-BMP, 50μM 2-BMP and 20μM MG-132 (Calbiochem), or an equal volume of DMSO before collection for Western blotting and immunofluorescence analysis. For Western blotting, 2-BMP, 2-BMP and MG-132, or DMSO treated schizont infected erythrocytes were lysed with 0.1% Saponin in PBS as described above and schizonts were solubilized in 1X Lamelli Sample Buffer with 5% β-mercaptoethanol. Western blotting of resulting lysates was performed as described in main experimental procedures. Densitometry analysis was performed using ImageJ, which is freely available at (http://rsbweb.nih.gov/ij/). For immunoflourescence, parasites were fixed in 4% paraformaldehyde/0.01% Gluteraldehyde in PBS and permeabilized with 0.1% TX-100 in PBS. Fixed parasites were stained with PfGAP45 or PfMSP1 antisera and then with the appropriate secondary antibody before being visualized on a Leica DM2500 microscope with a Leica DFC420C camera. Images were merged in Adobe Photoshop CS4.

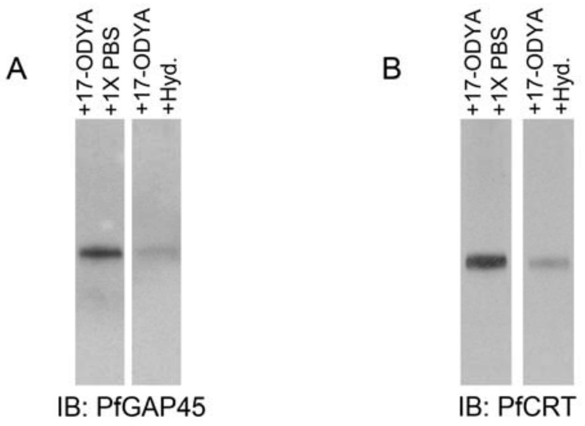

Figure S1. 17-ODYA Is Linked to Labeled Proteins via a Thioester Bond, Related to Figure 1

A. Anti-PfGAP45 antibodies were used to detect the purification of PfGAP45 after labeling of asexual-stage *P. falciparum* with 17-ODYA, click-chemistry, precipitation on streptavidin-agarose, and mock hydroxylamine (left lane; +17-ODYA and +PBS) or hydroxylamine treatment (right lane; +17-ODYA and +hydroxylamine) of agarose beads prior to biotinylated protein elution.
B. Anti-PfCRT antibodies were used to detect the purification of PfCRT after labeling of asexual-stage *P. falciparum* with 17-ODYA, click-chemistry, precipitation on streptavidin-agarose, and mock hydroxylamine (left lane; +17-ODYA and +PBS) or hydroxylamine treatment (right lane; +17-ODYA and +hydroxylamine) of agarose beads prior to elution of biotinylated proteins.

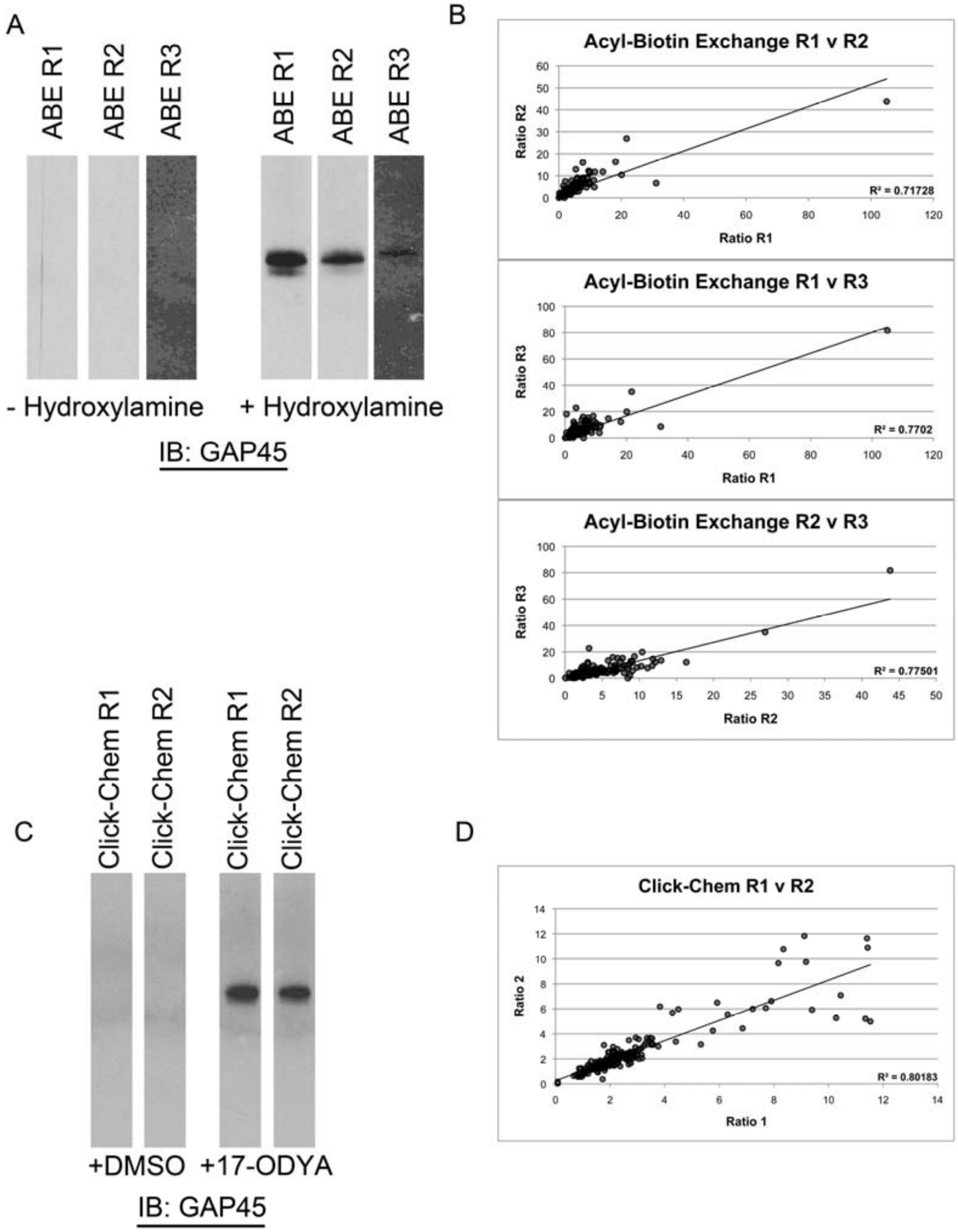

Figure S2. Palmitoyl-Proteins Were Purified in All Biological Replicates Used for Quantitative Mass Spectrometry Analysis and Results Were Reproducible, Related to Figure 2

A. A small aliquot of each ABE replicate was analyzed by Western blot for the purification of PfGAP45 before being mixed and processed for mass spectrometry analysis. Anti-PfGAP45 antibodies were used to detect PfGAP45 in all palmitome (right lanes; +hydroxylamine) and control samples (left lanes; -hydroxylamine).
B. ABE-generated MaxQuant ratios are reproducible. Presented here are scatter plot comparisons of all ratios resulting from a ratio count of at

least 2 for all proteins identified in biological ABE replicates. R-squared values reported in lower right corner.

C. A small aliquot of each metabolic labeling and click-chemistry replicate was analyzed by Western blot for the purification of PfGAP45 before being mixed and processed for mass spectrometry analysis. Anti-PfGAP45 antibodies were used to detect PfGAP45 is in all palmitome (right lanes; +17-ODYA) and control samples (left lanes; +DMSO).
D. Metabolic labeling and click-chemistry-generated MaxQuant ratios are reproducible. Presented here is a scatter plot comparison of all ratios resulting from a ratio count of at least 2 for all proteins identified in metabolic labeling and click-chemistry biological replicates. R-squared value reported in lower right corner.

A

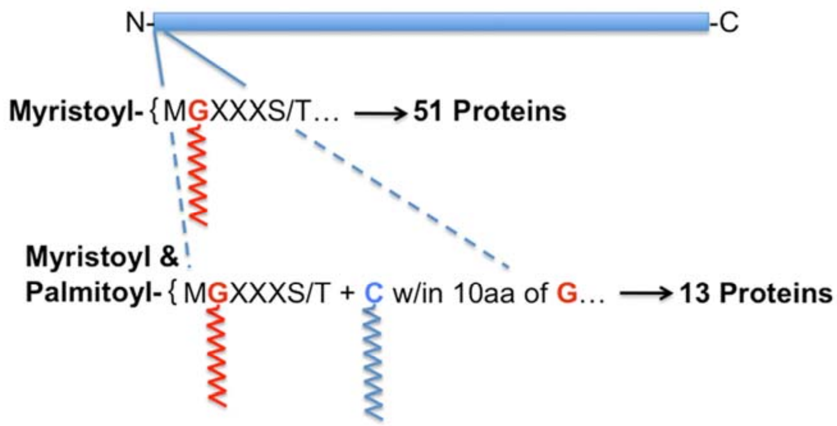

B

	Proteins with an N-terminal Dual-acylation Motif	Protein Name	Dual-acylation motif…	Max Time of Expression	ABE Max Mean Ratio	CC Max Mean Ratio
Present in Total Palmitome	PF10_0107	conserved protein, unknown function	MGNIVSCCSL…	Late Trophozoite	16.78	8.46
	MAL13P1.310	calpain	MGCINSKVKE…	Early Ring	10.19	
	PFL1090w	glideosome-associated protein 45	MGNKCSRSKV…	Late Schizogony	9.74	12.05
	MAL13P1.44	protein phosphatase 2c-like protein, putative	MGNCASVINH…	Late Schizogony	7.60	
	MAL8P1.109	protein phosphatase, putative	MGTCISFLKK…	Late Schizogony	6.09	
	PFB0815w	calcium dependent protein kinase 1	MGCSQSSNVK…	Late Schizogony	4.71	3.60
	MAL13P1.51	secretory complex protein 61 alpha,Rab GTPase 5b	MGCSSSTERL…	Late Schizogony	2.76	
	PF08_0064	vacuolar protein-sorting protein VPS2, putative	MGTYFSKDLQEC…	Gametocyte	1.83	2.27
Present in Proteome	PFI1005w	ADP-ribosylation factor, putative	MGNTVTTFFRDCCN…	Late Schizogony		
	PFL1110c	cAMP-dependent protein kinase regulatory subunit	MGNVCTWRQGK…	Early Schizogony		
Not Identified	PF08_0062	adenylate kinase 2	MGSCYSRKNKV…	Late Ring		
	PF11_0307	phosphatidylinositol-4-phosphate-5-kinase, putative	MGNKLTCGEI…	Gametocyte		
	PFI0675w	conserved Plasmodium protein, unknown function	MGCRLSKAND…	Late Schizogony		

Figure S3. The Majority of Predicted N-myristoyl and Palmitoyl-Proteins Are Identified by This Analysis, Related to Figure 3

A. Primary sequence-based identification of putative N-myristoyl-palmitoyl-proteins. *P. falciparum* predicted proteins were searched for the N-myristoylation motif, N-MGXXXS/T, resulting in the identification of 51 proteins (all TM-domain-containing proteins were discarded). These 51 proteins were manually searched for the presence of a cysteine residue within 10 amino acids of the putative N-myristoylation motif, resulting in the identification of 13 predicted N-myristoyl-palmitoyl-proteins.
B. Most putative N-myristoyl-palmitoyl-proteins are identified by the mass spectrometry analysis presented here. Proteins listed in top section of table are enriched by ABE and/or metabolic labeling and click chemistry. Proteins listed in middle section are found in the total proteome identified during this work, but are not enriched by either palmitoyl-protein purification method. Proteins in the last section are

not identified in the palmitome or in the proteome associated with this work. The protein name, putative dual-acylation motif, and time of maximum expression (as described on PlasmoDB) is detailed for each protein.

Protein ID	Protein Description	ABE Max Mean Ratio	CC Max Mean Ratio	Position	PEP	Sequence
MAL7P1.27	chloroquine resistance transporter	13.70	6.65	301	7.62E-03	NTVVEN**C(ca)**GLGMAK
PFL1090w	glideosome-associated protein 45	9.74	12.05	160	5.13E-13	SVTP**C(ca)**DMNKLDETAK
PF14_0578	conserved Plasmodium protein, unknown function	9.41	10.21	96	4.05E-50	NS**C(ca)**ETLLESEQNLLKSPK
PF10_0187	60S ribosomal protein L30e, putative	3.01	2.00	51	9.73E-12	LVIVSSN**C(ca)**PSIQR
PFI1090w	S-adenosylmethionine synthetase	2.06	2.81	113	1.71E-06	VSIDEQSPDIAQ**C(ca)**VHENR
PFI1105w	phosphoglycerate kinase	1.64	2.83	98	1.05E-21	GLLGEEVLFLND**C(ca)**VGK
MAL13P1.214	phosphoethanolamine N-methyltransferase	1.46	2.94	70	2.47E-48	VLDIGSGLGGG**C(ca)**MYINEK
MAL8P1.69	14-3-3 protein, putative	1.46	2.36	149	2.43E-05	YISEFS**C(ca)**DEGKK

Figure S4. Several Palmitolylation Sites Are Identified in Peptides Isolated from Proteins Present in the Total Palmitome, Related to Figure 5

PEP (Posterior Error Probability) values are listed for each peptide, and carbamidomethylated cysteines (palmitoylation sites) are highlighted in bold and designated with (ca).

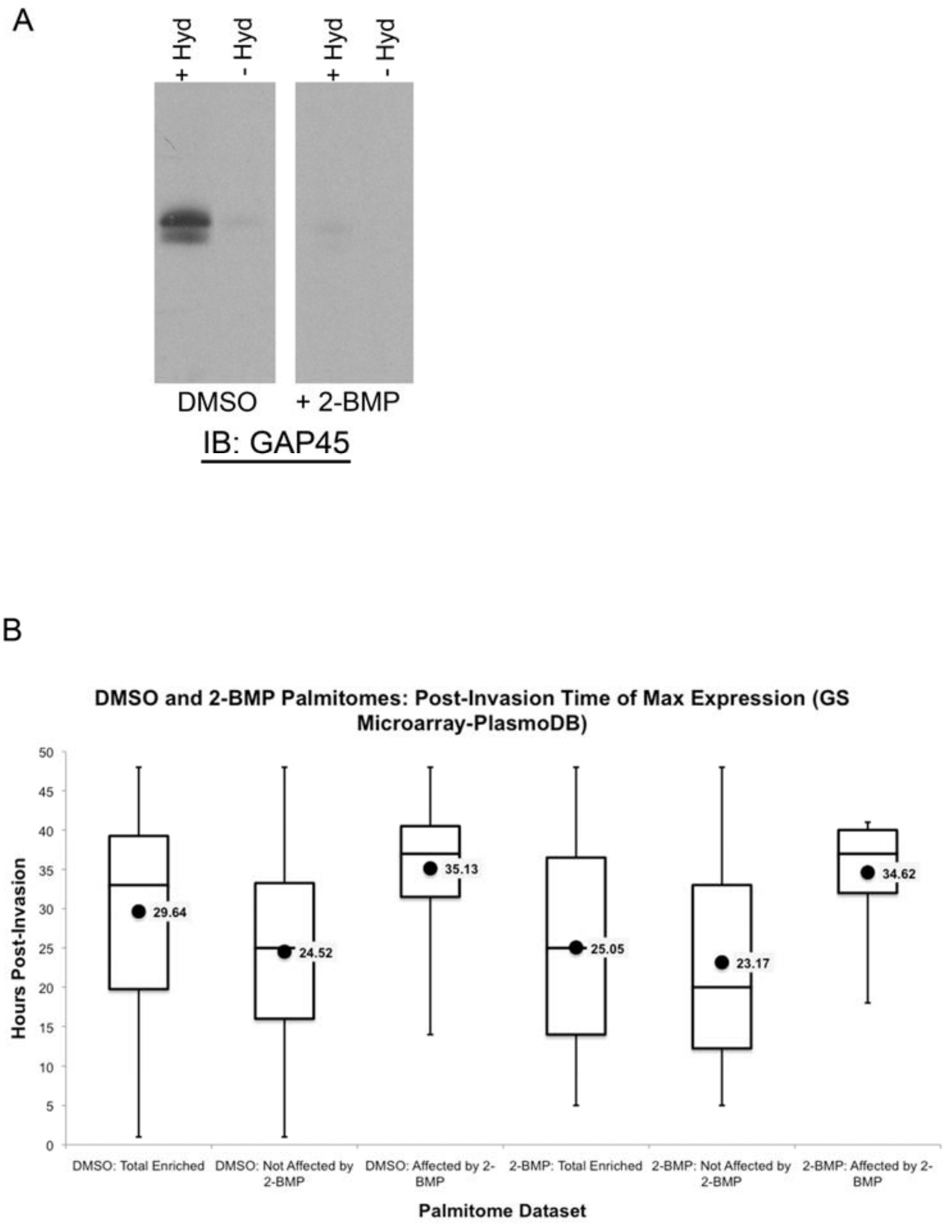

Figure S5. PfGAP45 Is Not Purified by ABE after 2-BMP Treatment and the Palmitome Purified from 2-BMP-Treated Asexual-Stage *P. falciparum* Is Composed of Proteins Expressed at an Earlier Time in Development, Related to Figure 6

A. A small aliquot of ABE eluates (+ or – hydroxylamine) prepared using DMSO (left lanes) or 2-BMP-treated (right lanes) schizonts were analyzed by Western blot with anti-PfGAP45 antibodies to detect PfGAP45 before being processed for quantitative mass spectrometry analysis.
B. Time of maximum expression data (PlasmoDB) were used to determine when proteins enriched in DMSO or 2-BMP palmitomes were generally transcribed. These data were also used to determine when proteins affected by 2-BMP treatment generally reached their

point of maximum expression. Plot displays the minimum value (lower whisker), maximum value (upper whisker), 2nd (lower box) quartile, and 3rd (upper box) quartile of the post-invasion time of maximum expression (transcription) for the groups of proteins defined on the X-axis. Mean value for hours-post-invasion time of maximum expression is noted for each group. Timing of expression data taken from PlasmoDB (GS-array; (Bozdech et al., 2003)).

Supplemental References

Boersema, P.J., Raijmakers, R., Lemeer, S., Mohammed, S., and Heck, A.J. (2009). Multiplex peptide stable isotope dimethyl labeling for quantitative proteomics. Nat Protoc *4*, 484-494.

Bozdech, Z., Llinas, M., Pulliam, B.L., Wong, E.D., Zhu, J., and DeRisi, J.L. (2003). The transcriptome of the intraerythrocytic developmental cycle of Plasmodium falciparum. PLoS Biol *1*, E5.

Cox, J., and Mann, M. (2008). MaxQuant enables high peptide identification rates, individualized p.p.b.-range mass accuracies and proteome-wide protein quantification. Nat Biotechnol *26*, 1367-1372.

Cox, J., Neuhauser, N., Michalski, A., Scheltema, R.A., Olsen, J.V., and Mann, M. (2011). Andromeda: a peptide search engine integrated into the MaxQuant environment. J Proteome Res *10*, 1794-1805.

Nkrumah, L.J., Muhle, R.A., Moura, P.A., Ghosh, P., Hatfull, G.F., Jacobs, W.R., Jr., and Fidock, D.A. (2006). Efficient site-specific integration in Plasmodium falciparum chromosomes mediated by mycobacteriophage Bxb1 integrase. Nat Methods *3*, 615-621.

Theron, M., Hesketh, R.L., Subramanian, S., and Rayner, J.C. (2010). An adaptable two-color flow cytometric assay to quantitate the invasion of erythrocytes by Plasmodium falciparum parasites. Cytometry A *77*, 1067-1074.

Wessel, D., and Flugge, U.I. (1984). A method for the quantitative recovery of protein in dilute solution in the presence of detergents and lipids. Anal Biochem *138*, 141-143.
